# Intestinal *in vitro* and *ex vivo* Models to Study Host-Microbiome Interactions and Acute Stressors

**DOI:** 10.3389/fphys.2018.01584

**Published:** 2018-11-12

**Authors:** Sarah C. Pearce, Heidi G. Coia, J. P. Karl, Ida G. Pantoja-Feliciano, Nicholas C. Zachos, Kenneth Racicot

**Affiliations:** ^1^Performance Nutrition Team, Combat Feeding Directorate, Natick Soldier Research, Development and Engineering Center, Natick, MA, United States; ^2^National Research Council, The National Academies of Sciences, Engineering, and Medicine, Washington, DC, United States; ^3^711th Human Performance Wing, Airforce Research Laboratory, Airman Systems Directorate, Human-Centered ISR Division, Molecular Mechanisms Branch, Wright-Patterson Air Force Base, Dayton, OH, United States; ^4^Military Nutrition Division, U.S. Army Research Institute of Environmental Medicine, Natick, MA, United States; ^5^Soldier Protection and Optimization Directorate, Natick Soldier Research, Development and Engineering Center, Natick, MA, United States; ^6^Division of Gastroenterology and Hepatology, Department of Medicine, Johns Hopkins University School of Medicine, Baltimore, MD, United States

**Keywords:** physiology, stress, *ex vivo*, *in vitro*, intestine, DoD

## Abstract

The gut microbiome is extremely important for maintaining homeostasis with host intestinal epithelial, neuronal, and immune cells and this host-microbe interaction is critical during times of stress or disease. Environmental, nutritional, and cognitive stress are just a few factors known to influence the gut microbiota and are thought to induce microbial dysbiosis. Research on this bidirectional relationship as it pertains to health and disease is extensive and rapidly expanding in both *in vivo* and *in vitro/ex vivo* models. However, far less work has been devoted to studying effects of host-microbe interactions on acute stressors and performance, the underlying mechanisms, and the modulatory effects of different stressors on both the host and the microbiome. Additionally, the use of *in vitro*/*ex vivo* models to study the gut microbiome and human performance has not been researched extensively nor reviewed. Therefore, this review aims to examine current evidence concerning the current status of *in vitro* and *ex vivo* host models, the impact of acute stressors on gut physiology/microbiota as well as potential impacts on human performance and how we can parlay this information for DoD relevance as well as the broader scientific community. Models reviewed include widely utilized intestinal cell models from human and animal models that have been applied in the past for stress or microbiology research as well as *ex vivo* organ/tissue culture models and new innovative models including organ-on-a-chip and co-culture models.

## Introduction

The mammalian intestine is critically important for nutrient digestion and absorption, ion and water transport, as well as maintaining a homeostatic relationship with immune cells and the trillions of bacteria that reside there collectively known as the gut microbiome. The gut microbiota consists of not only bacteria, but also viruses, fungus, yeast, and archaea. *Bacteroidetes* and *Firmicutes* phyla make up ~90% of the human adult gut microbiota but there is a wide range of species diversity numbering in the hundreds to thousands (Macfarlane and Macfarlane, 2004; Eckburg et al., 2005). Bacterial diversity within the human microbiota demonstrates inter-individual variability, and can be influenced by the environment, genetics, diet, antibiotic use, and geographical location (Zhu et al., 2015). Normally, the gut microbiome significantly contributes toward protection against pathogens by competing for shared nutrients and niches or through enhancing host defense mechanisms (Kinross et al., 2011).

A balanced host-microbe interaction is necessary for maintaining homeostasis. Intestinal epithelial cells can sense and respond to the microbial environment by secreting signaling compounds, such as cytokines or chemokines as well as anti-microbial peptides and hormones, reflecting its notoriety as an endocrine organ (Smirnova et al., 2003; Clarke et al., 2014). The normal intestinal epithelium consists of several cell types including enterocytes, goblet cells, stem cells, enteroendocrine cells, Tuft cells, M cells and Paneth cells, all of which can sense and respond to bacteria by several means including producing and secreting anti-microbial peptides (Paneth cells, enterocytes), producing and secreting mucin (goblet cells), as well as secreting cytokines, and expressing toll-like receptors (TLR's) and nod-like receptors (NLR's). The epithelium of the small intestine consists of a single mucus layer that acts as a protective barrier between cells and bacteria whereas in the colon there is a double mucus layer consisting of a sterile, tightly-adhered mucus layer and a loose layer which provides a niche for bacteria (Johansson et al., 2008). This mucus layer produced by mucin-secreting goblet cells can influence the types of bacteria that reside in the gut (Van den Abbeele et al., 2013).

Aside from epithelial cell interactions, the gut microbiome also interacts with the host immune system largely through gut-associated lymphoid tissue (GALT) system (Doe, 1989; Mowat, 2003; Round et al., 2010; Hooper et al., 2012) and in some ways the immune system is actually “educated” by the gut microbiota to distinguish friend or foe. The gut comprises the largest lymphoid system in the human body. The GALT is separated from the lumen by the epithelial cell layer. Underneath this epithelial layer is an underlying layer called the lamina propria as well as lymphatic circulation that can house several different types of immune cells including macrophages, mast cells, and plasma cells. In the small intestine there are Peyer's patches that consist of isolated lymphoid follicles that house B cells, T cells and dendritic cells (Doe, 1989; Mowat, 2003). The host is equipped with pattern recognition receptors (PRR's) that recognize pathogen-associated molecular patterns (PAMPs); recognition of these PAMPS leads to immune activation and production of anti-microbial peptides and cytokines/chemokines. As briefly mentioned earlier, PRR's include the family of TLRs and NLR's that recognize bacterial and viral ligands. Epithelial cells are capable of producing various anti-microbial peptides (AMPS) including defensins and c-type lectins. These systems are designed to defend against pathogens but in some cases are also thought to be associated with auto-immunity (van Kooyk, 2008; Frasca and Lande, 2012).

The enteric nervous system (ENS) is also important for maintaining homeostasis and is involved in the host-microbiome response. The ENS is a large neural network embedded in the tissue of the GI tract and has been referred to as the body's “second brain.” It helps with peristalsis in the gut, hormone secretion, neurotransmitter release, and signaling to the central nervous system (CNS) (Zhu et al., 2017). It is also known that stress and the microbiota can have effects on the ENS (Mayer et al., 2015) affecting both GI function as well as CNS function. Also, the gut microbiome has an important relationship with the endocannabinoid system, which is a complex system involved in energy homeostasis and metabolism (Cani et al., 2014). Enteroendocrine cells are the most numerous endocrine cells in the human body that produce and secrete gastrointestinal hormones/peptides, and are involved in appetite regulation. Although they are the most numerous type of endocrine cells, they represent < 1% of all intestinal epithelial cells. Additionally, these cells are known chemosensors that interact with the host immune system and express several functional TLR's (Yoo and Mazmanian, 2017). Although there has been increasing evidence that the microbiome influences the gut-brain axis; little *in vitro*/*ex vivo* work has examined this potential link, thus this topic will not be discussed in detail in this review (Carabotti et al., 2015).

The epithelial layer works in conjunction with immune cells, smooth muscle layer, and nerve cells in a coordinated effort to promote homeostasis. The host-microbiome interaction is largely symbiotic and mutualistic in nature. However, perturbations in this homeostasis can lead to dysbiosis, decreased intestinal barrier function, nutrient malabsorption/diarrhea, infection (sepsis), and/or auto-immune disorders (Aleksandrova et al., 2017). Dysbiosis is characterized by a compositional shift from obligate anaerobes to facultative anaerobe classes within the gut microbiome community (Winter and Baumler, 2014). Specifically, the gut microbiome plays a large role in maintaining gut health by metabolizing nutrients to create short chain fatty acids (SCFA) and other beneficial metabolites, such as polyphenols to the host (Russell et al., 2013). In addition, bacteria normally found in the mammalian gut, synthesize vitamins including Vitamin K and Vitamin B12 (LeBlanc et al., 2013). The gut microbiota is also involved in intestinal barrier defense, and priming the immune response (Elson and Alexander, 2015) and interact with the neuroendocrine system (Farzi et al., 2018). Various conditions and environments have been shown to alter microbial composition and cause decreased epithelial barrier function, and inflammation, such as intense exercise/training, hot or cold environments, high altitude, sleep deprivation, caloric deprivation, psychological stress and changes in diet. However, there is evidence to suggest that the resilience of the gut microbiome may positively or negatively impact health (Sommer et al., 2017).

Studying these complex-interactions *in vitro* and *ex vivo* is a constantly evolving area. Therefore, this review addresses the current knowledge along with strengths and limitations of intestinal models utilized in the study of host-microbiome interactions. This review will pertain mainly to gut models and mechanisms of bacteria-epithelial interactions, although where appropriate will also include co-culture models that mimic the gut-immune or gut-brain axes.

## Military relevance

Military training and combat is frequently characterized by exposure to various stressors. These stressors are psychological (e.g., fear, anxiety, trauma, cognitive demands), environmental (e.g., heat, cold, high altitude, pathogens), and physical (e.g., strenuous exercise, undernutrition, sleep deprivation) in nature, and are often experienced at extremes and in combination (Weeks et al., 2010; Henning et al., 2011). Associated physiological effects are myriad (Nindl et al., 2013), and affect every organ system in the body including the nervous and immune systems and gastrointestinal tract. For example, it is well-established that hypoxic (i.e., high altitude), heat and cold stress cause changes in cognition, appetite, intestinal function and permeability, and immune function (Saunders et al., 1994; Coskun et al., 1996; Anand et al., 2006; Lambert, 2008; Zhou et al., 2011; Pearce et al., 2014; Dokladny et al., 2016; Khanna et al., 2017). For instance, cold exposure, which is a period of high energy demand, can shift the composition of the gut microbiota that can favor energy extraction and intestinal absorptive surface (Chevalier et al., 2015). In military personnel, these effects are often compounded by sleep deprivation and concomitant increases in physical exertion and inadequate dietary intakes (Friedl et al., 1994; Montain and Young, 2003; Tharion et al., 2005; Margolis et al., 2014, 2016; Karl et al., 2017b) that independently influence nervous system, immune, and gastrointestinal function (Everson and Toth, 2000; Lambert, 2008; Li et al., 2013, 2014; Zuhl et al., 2014; Schmid et al., 2015; Clark and Mach, 2016; Karl et al., 2017a). In deployed settings in particular, traumatic psychological events (Creamer et al., 2011; Bonde et al., 2016) and exposure to enteric pathogens (Porter et al., 2017; Riddle et al., 2017) are additional stressors potentially impacting nervous system, immune, and gastrointestinal function (Lafuse et al., 2017; Mackos et al., 2017). A summary of effects of military-relevant stressors on the gut microbiota was recently published by Karl et al. (2018).

Growing evidence from animal, and, to a far lesser extent, human studies indicates that stressor-induced changes in nervous system, immune, and gastrointestinal function alter the gut microbiome, and the gut microbiome, in turn, alters physiologic and cognitive responses to these stressors (Kleessen et al., 2005; Zhou et al., 2011; Holmes et al., 2012; Galley and Bailey, 2014; Chevalier et al., 2015; David et al., 2015; Clark and Mach, 2016; Thaiss et al., 2016; Voigt et al., 2016; Zietak et al., 2016; Allen et al., 2017; Mackos et al., 2017). This suggests that the gut microbiota could be an underappreciated mediator of stress responses and associated outcomes in military personnel. Indeed, gut microbiota composition and activity has recently been associated with gastrointestinal permeability, inflammation, gastrointestinal symptoms, and psychological metrics during military training (Li et al., 2013, 2014; Phua et al., 2015; Karl et al., 2017a,b). Elucidating mechanisms by which host-gut microbiome interactions impact host stress responses could therefore facilitate development of gut microbiome-targeted interventions for health and performance optimization in military personnel. Of note, potential benefits of such interventions extend beyond the military as military-relevant stressors are not uncommon, alone or in combination, in some civilian populations, such as athletes (Clark and Mach, 2016) and first responders, such as firefighters (Alexander and Klein, 2009).

Our current understanding of the mechanisms underpinning host-gut microbiome interactions is largely derived from rodent studies, particularly germ-free mice. However, the relevance of these models to human host-gut microbiome interactions has been questioned (Nguyen et al., 2015). Moreover, many of these studies have focused on chronic health and disease rather than shorter-term outcomes. From a military and athletic perspective, elucidating mechanisms of host-gut microbiome interactions and how they can be leveraged to build resiliency, and optimize physical and cognitive performance under transient stress exposure is perhaps of greater interest. *In vitro* and *ex vivo* models have become an integral tool in examining mechanisms underlying host-pathogenic and host-commensal bacterial interactions. As such, although many of the *in vitro* and *ex vivo* models reviewed below have been used for examining mechanisms of disease, it is important to consider how these models can be applied to study the complex interplay between stress exposure, the gut microbiome, and host physiology.

## Intestinal host models for microbiome research

### In vitro

#### Caco-2

Developed in the 1970's, the Caco-2 cell line has been widely studied in the pharmacological, nutritional, and microbiological fields. This cell line was derived from human colorectal adenocarcinoma and can either function as undifferentiated large intestinal cells, or can spontaneously differentiate to resemble a small intestine-like phenotype with enterocyte-like absorptive properties. A brush border expressing clone of these cells (Caco-2 BBe) has also been produced to act as a small intestinal mimic. Due to the spontaneity of this cell line, it has been difficult to control and reproduce data; however it is desirable for studying transport kinetics and can act as a small or large intestine mimic (Shi et al., 2017). Over 200 papers have examined bacteria and bacterial metabolites using the Caco-2 model to study intestinal barrier function, bacterial adhesion/invasion, and innate immune response. Beneficial and pathogenic bacteria have been used to treat Caco-2 cells and elucidate protective effects of *Lactobacillus rhamnosus GG* and *Lactobacillus casei* against inflammation (Toki et al., 2009). This study found that *L. rhamnosus GG* and *L. casei* do not induce pro-inflammatory cytokine expression and actually suppress certain cytokines induced by *E. coli*, or bacterial ligands. Caco-2 cells have also been shown to express functional toll-like receptors that can respond following bacterial challenge (Furrie et al., 2005). Interestingly, Caco-2 cells have also been used to study prebiotic oligosaccharides. Prebiotic oligosaccharides have been shown to positively modulate the intestinal microbiota and Caco-2 cells treated with oligosaccharides exhibit reduced inflammatory activity through the PPARγ pathway (Zenhom et al., 2011). A comprehensive and eloquent review on human colon cancer cell lines that discusses mechanisms of pathogenesis of human enterovirulent bacteria was written in 2013 that also encompasses the following two cancer cell lines (Lievin-Le Moal and Servin, 2013).

#### HT-29

HT-29 is another polarized, but largely undifferentiated, human colorectal adenocarcinoma that was isolated in the 1970's. Since this time, several sub-populations have been created to be more “enterocyte-like.” HT-29 cells were originally used to study cancer biology but were transitioned over to other research due to their phenotype (Rousset, 1986). HT-29 cells can now be grown under a non-polarized, undifferentiated state or be cultured to differentiate and obtain a polarized membrane depending on the objective. This cell line also produces cytokines, such as interleukins, TNF-α (De Simone et al., 2015; Khan Mirzaei et al., 2016). For this reason they have been extensively studied in the field of nutrition and host-microbiome interactions. Like Caco-2, using a Transwell® system with apical and basolateral polarity allows for studies of bacterial adhesion to the epithelium, as well as bacterial transport. This model is unique compared to Caco-2 in that HT-29 cells contain mucus-producing goblet cells and for that reason they have also been used as models to study immune function and bacterial-host interactions with greater success (Raja et al., 2012; Altamimi et al., 2016). For example, this cell line has been a useful tool to show that oligosaccharides and a functional mucin layer can reduce bacterial adhesion to the epithelium (Altamimi et al., 2016). This cell line has been well-characterized in regards to host response to bacterial infection including adhesion, migration and internalization of pathogens including *Salmonella, E. coli*, and others. HT29-MTX cells, which are HT-29 cells treated with methotrexate, express higher proportions of mucin-secreting goblet cells and have been utilized to study bacterial survival and adhesion (Dahiya et al., 1992). Mucins expressed in this line include both secretory (MUC2, MUC5AC, MUC6) as well as membrane bound (MUC1, MUC3, MUC4) types (Huet et al., 1995). Researchers have co-cultured Caco-2 and HT-29 to make the model more physiologically and functionally relevant, especially for the study of microbiology. HT-29 cells in combination with Caco-2 have been used to study protective effects of probiotics against pathogenic bacteria (Resta-Lenert and Barrett, 2003) by showing that pre-exposure of epithelial cell monolayers to live probiotic *Streptococcus thermophilus* and *Lactobacillus acidophilus* limits invasion and adhesion of enteroinvasive *Escherichia coli* (EIEC). Interestingly, HT-29 cells have become a host model to test *in vitro* fermentation of prebiotics (Maccaferri et al., 2012) and fecal fermentation. One study examined modulation of immune response by two different probiotic *Bifidobacterium* species in a single-stage continuous-culture system combined with epithelial cells (Arboleya et al., 2015). This study showed that HT-29 cells combined with fermentation, can be a tool to screen different bacterial species probiotic potential. It showed that. For these to be an effective model to utilize for host-microbiome interactions, they need to have a viable mucin layer.

#### T84

Similar to Caco-2 and HT-29, T84 cells were derived from human colon cancer cells. However, unlike the former two lines, T84 cells were isolated from metastasized cells found in the lungs. T84 cells express several brush border properties including brush border enzymes and transporters. These have been extensively utilized as a model for studying epithelial barrier function and electrolyte transport as they attain tightly formed tight junctions and express tight junction proteins including claudins, occludin, and ZO-1, as well as ion transporters (Madara and Dharmsathaphorn, 1985). These cells were initially used to study heat-stable *E. coli* enterotoxin. These toxins induce intestinal secretion but the mechanisms were not previously understood. T84 cells were utilized to study binding of the enterotoxin, activation of guanylate cyclase and cGMP production to provide new information on the mechanism of action (Guarino et al., 1987). T84 cells have also been expanded to study probiotic bacterial supernatants and their protective effects during EIEC invasion. Results determined that probiotics may produce beneficial metabolites that can prevent bacterial invasion (Khodaii et al., 2017). This has been one of the lesser utilized models for microbiome research however researchers have still found value in this model to study bacterial interactions, especially when conducting experiments in parallel with other related cell lines for a more robust outcome. An interesting new study was published using T84 cells to elucidate the effects of a microbial-derived protein by-product and its ability to maintain intestinal barrier integrity. Tryptamine was able to reduce inflammatory cytokine-induced monolayer permeability. In addition, indole-3-propionic acid (IPA) had positive effects on fructose metabolism (Jennis et al., 2018). Additionally, T84 cells have been utilized to study protective effects of Lactoferrin on bacterial-induced barrier dysfunction showing that Lactoferrin maintains tight junction structure during *Yersinia enterocolitica* infection (Hering et al., 2017).

#### IEC-6 and IEC-18

IEC-6 and IEC-18 are lines derived from the small intestine of rats. IEC-6 is derived from the whole small intestine while IEC-18 is ileum derived. As these lines are small intestinal, they do not accurately reflect colonic morphology and physiology; however, they have become a useful tool to study bacterial adhesion (Cinova et al., 2011). Adhesion of multiple bacterial strains including *E. coli* CBL2 and *Shigella* CBD8 were tested using IEC-6 cells. IEC-6 was initially utilized to study transport of microflora-derived products (Osborne and Seidel, 1989) and attachment of *Giardia intestinalis* (McCabe et al., 1991) due to its similarity to primary cultures and previous lack of a convenient *in vitro* model. These cells, like many of the lines, express cytokines, such as interleukin-6 as well as toll-like receptors (Li et al., 2011). IEC-6 has also been utilized to study small-intestinal microbiota-related bacteria. More recently it has been used to examine potential probiotics including *Bifidobacterium bifidum* (Khailova et al., 2010) and *Lactobacillus reuteri* (Liu et al., 2010). IEC-6 cells have been able to show protective effects against LPS-induced intestinal inflammation using *L. reuteri* (Liu et al., 2010). Additionally, *Escherichia coli Nissle* 1,917 supernatants were shown to be effective in reducing apoptosis, and improving barrier function during simulated epithelial damage (Wang et al., 2014). IEC-18 cell lines have been used to examine protective effects of *Bifidobacteria spp*. against injury through enhancement of intestinal barrier function and alterations in TLR signaling (Yang et al., 2017). IEC-18 cells have also been shown to have bactericidal capabilities (Deitch et al., 1995).

#### IPEC-J2 and IPEC-1

The IPEC cell lines are porcine intestinal epithelial cells isolated from neonatal piglet small intestine (Rhoads et al., 1994). IPEC-J2 are jejunum derived while IPEC-1 are from the jejunum and ileum. IPEC-J2 and its usefulness in microbiological investigations was more thoroughly investigated in 2011 (Brosnahan and Brown, 2012). It is unique to most of the other polarized cell lines in that it is non-transformed, and not of cancerous origin. In addition, pig intestine much more closely resembles human intestine compared to mice or rodent models. IPEC cells express tight junction proteins (Schierack et al., 2006), multiple mucins, and are able to express and secrete many types of cytokines/chemokines. They also express inflammatory pathway markers NFKB and MyD88 (Mariani et al., 2009), and toll-like receptors (Arce et al., 2010) that are necessary for assessing host-microbe interactions. They have been utilized to study bacterial infections that affect both swine as well as humans due to physiologic similarities.

The first use of IPEC-J2 cells in microbiological studies involved the pathogenic bacteria *Lawsonia intracellularis* (McOrist et al., 1995). This study showed that the *in vitro* culture model closely resembled that of *in vivo* infection including mechanism release of internalized bacteria into the cytoplasm and release of bacteria from the epithelial monolayer. They have been largely utilized to study innate immune responses to various pathogens including *Salmonella enterica* (Schierack et al., 2006), *S. typhimurium* (Boyen et al., 2009), *E. coli*, as well as Chlamydia (Schierack et al., 2006). In these studies, all infected IPEC-J2 cells encoded mRNA's for several cytokines including IL6, TNF-alpha, and IL1-alpha. *Salmonella* alone was shown to enhance IL-8 expression. Additionally IPEC-J2's have been increasingly used for probiotic research. Adhesion abilities of 11 strains of *Lactobaillus* spp. were analyzed using IPEC-J2 cells, determining that *L. reuteri* and *L. plantarum* have the highest binding capacities (Larsen et al., 2007). *Bacillus licheniformis* was shown to induce IL8 mRNA expression. However, when co-cultured with *Salmonella enterica, B. licheniformis* inhibits basolateral IL8 secretion. (Skjolaas et al., 2007). *Bifidobacterium* was shown to have anti-viral activity including inhibition of viral invasion of host cells, and production of anti-viral metabolites (Botic et al., 2007). These cells have also been utilized to study cell proliferation, nutrient and ion transport, viral infection (rotavirus, vesicular stomatitis virus) and fungal infection (toxin from *Fusarium* fungus).

## Advantages and disadvantages of current *in vitro* models

There is an ongoing discussion amongst researchers on when to utilize primary cell models and when to utilize established cell lines. Transformed cell lines have long been considered the most cost-effective and enduring tool in basic research as they can be passaged indefinitely and are indispensable for preliminary screening and mechanistic studies. Primary cell lines are often considered to be more biologically and physiologically similar to *in vivo* models. Caco-2 cells grown as confluent monolayers are extremely useful to study absorptive and transport kinetics, especially in the drug transport field, under basal and bacteria exposed conditions. The main drawbacks of this cell line are its cancer cell origins, its homogeneity, and the fact that Caco-2 cells do not produce significant amounts of mucins under normal growth conditions (Pan et al., 2015). HT-29 cells contain a mucus layer that is useful for microbiome research, especially when treated with methotrexate. They have similar drawbacks to Caco-2 however, in that they are transformed cells from colon cancer and they are not normally utilized to study barrier function due to their inability to form proper tight junctions. T84 has very similar advantages and disadvantages to Caco-2 and HT-29 due to its ability to be grown as monolayers and its cancer origins but conversely to HT-29, T84 has been an excellent model to examine effects of microbes and stressors on epithelial barrier function due to its high TER properties. Overall, to utilize these cancer cell lines one must take into account several factors including culture conditions (media formulation, differentiated vs. undifferentiated), passage number, potential HeLa cell contamination, and whether they express the desired genes/proteins before determining whether these will be suitable for microbiological investigations.

IEC-6 cells are non-transformed and recapitulate normal small intestinal physiology. However, disadvantages are that it may not accurately reflect a human response in metabolism and absorption or colon physiology which is needed to study microbiology. Additionally, it is more useful to study small intestinal physiology and translatability from rodent to human is more difficult. IPEC-J2 cells are of porcine origin, and most similar to humans compared to other animal cell lines. These cells are non-transformed and mimic normal intestinal physiology and function. IPEC's are able to be polarized and contains multiple cell types, including mucus producing cells. However, its small intestinal origins make it difficult to translate to colon research. Also, like the other lines, media formulation and culture conditions can change the phenotype of the cell line.

In conclusion, *in vitro* models to date have not readily provided a non-cancerous colon model that accurately recapitulates a healthy large intestine but these models do have relevance depending on the application. Overall drawbacks of the above cell types are the lack of cellular diversity in a single cell type system. The normal intestinal epithelium consists of several cell types including enterocytes, goblet cells, stem cells, enteroendocrine cells, Tuft cells, M cells and Paneth cells that are not accurately represented. Additionally, translatability is also a concern in terms of species differences as well as cancerous vs. non-cancerous. They also lack the ability to culture bacterial community dynamics and are only able to study single bacterium-host interaction.

### Ex-vivo

#### InTESTine™ system by TNO

*Ex vivo* models are models cultured outside of an organism, but contain functional live tissues with complex cellular environments found *in vivo*. TNO, who makes the TIM-1, TIM-2, and TinyTim models of digestion and absorption, has recently developed a new *in vitro* system called the InTESTine™ that utilizes fresh healthy porcine intestinal tissue from multiple segments of the GI tract in parallel. This model was originally intended for drug discovery research but is meant to work in the presence of absence of microbiota. It is a more sophisticated model from the TIM systems as it incorporates actual intestinal tissue as opposed to mimicking conditions of the intestinal tract. This model contains a mucus layer that enables it to be more successfully utilized in conjunction with single or mixed communities of bacteria (TNO). Thus, far it has not been used in published microbiological investigations, but may provide a new way to study host-microbe interactions.

#### Ussing chamber

The Ussing chamber was initially developed by Hans Ussing to study transport across a variety of epithelial tissues, and has become a powerful *ex-vivo* tool for studying transport across different segments of the intestine which is a major advantage. It consists of two halves separated by polarized epithelia (tissue or cell monolayers) and the chamber is set up to isolate apical and basolateral sides. It works using electrodes that can measure voltage and short-circuit current to determine permeability/transport. This system has been utilized to study bacterial-host interactions largely via bacterial toxins, but also intact bacteria. The first such study examined the effects of *Shigella* enterotoxin on intestinal ion transport and showed that *Shigella* increases fluid and electrolyte accumulation, as well as net sodium secretion (Donowitz et al., 1975). Additionally, this technique was utilized to show that pathogenic *E. coli* increased Cl^−^ secretion by the intestinal epithelium (Hecht et al., 1999). Colonic mucosa has been mounted on Ussing chambers to study the effects of *Enterococcus faecalis* to look at bacterial invasion (Isenmann et al., 2000). This study determined that an aggregation substance (a bacterial adhesion product of *E. faecalis*) promotes bacterial translocation into colonic mucosa. *Clostridium difficile* was also tested in a Ussing chamber to test the hypothesis that anaerobic *C. difficile* interactions with host epithelial cells involve bacterial and toxin-mediated cellular events (Jafari et al., 2016). *Campylobacter jejuni* infection has been examined in this system using human colonic monolayers. *C. jejuni* was able to translocate across monolayers and cause an increase permeability by disruption of tight junctions. Other effects included increased release of lactate dehydrogenase, IL8, and prostaglandin E_2_ (Beltinger et al., 2008).

Potentially beneficial bacteria have also been studied in the Ussing chamber system including commercially available probiotic blend *Bifco* (Shi et al., 2014) and *Lactobacillus plantarum* (Chen et al., 2010). Bifico was able to improve epithelial barrier function, enhance resistance to EIEC infection, and reduce proinflammatory cytokine secretion. L. plantarum was also demonstrated to improve colonic epithelial barrier dysfunction in IL-10 knockout mice, by modulating epithelial junctions and PepT1-mediated transepithelial transport. The Ussing chamber model has been useful in demonstrating the ability of probiotics to promote intestinal barrier function.

#### Intestinal enteroids and organoids

**Figure 1:**
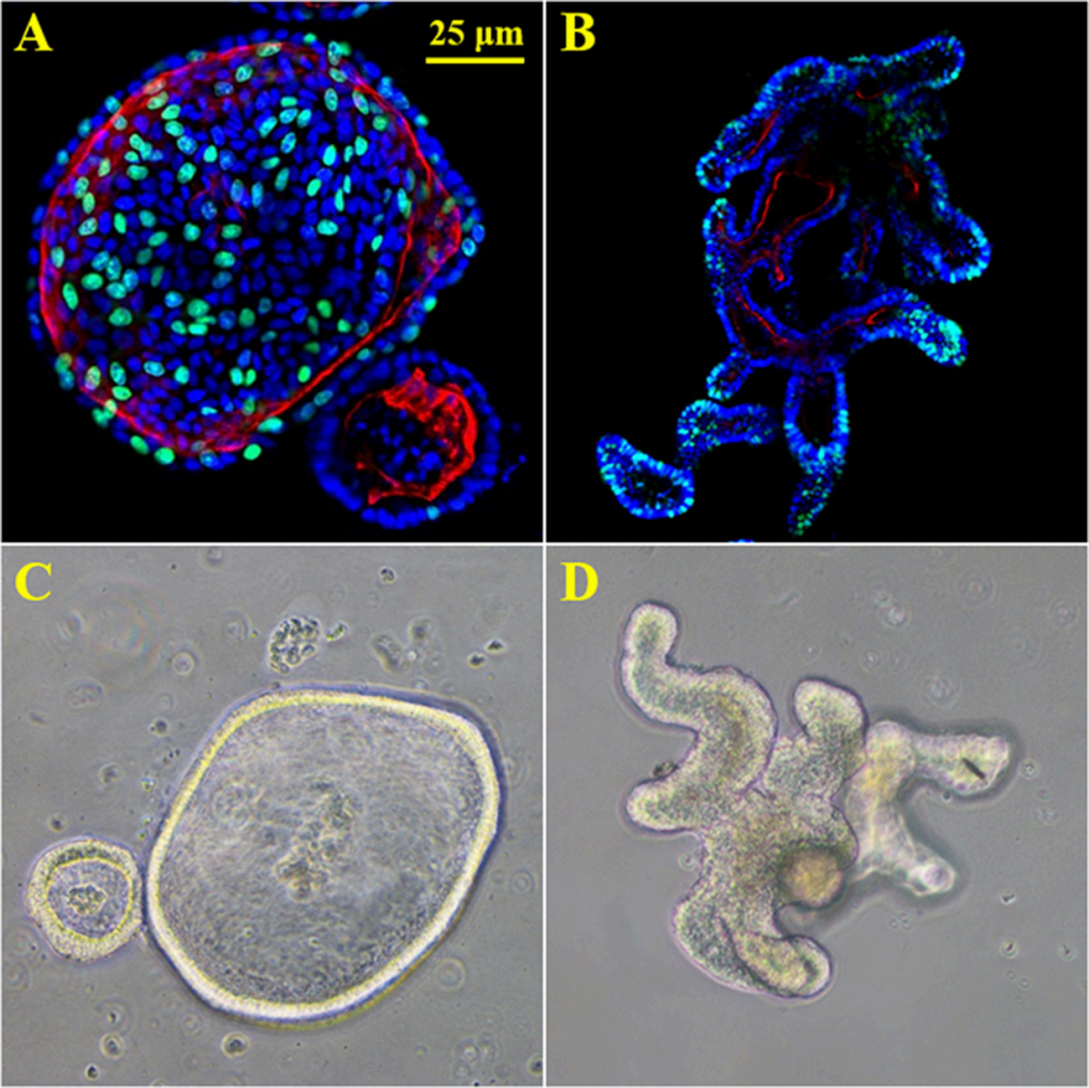
3D enteroids from human and mouse biopsy samples. **(A)** proliferation stain Edu (green), nuclei (blue) and f-actin (red) in human duodenal organoids. **(B)** proliferation stain Edu (green), nuclei (blue) and f-actin (red) in murine duodenal organoids. **(C)** 20X brightfield image of human duodenal organoids. **(D)** 20X brightfield image of murine duodenal organoids.

Organ culture of intestinal tissue was first described in 1969 by Browning and Trier (1969). This method utilized biopsy tissue and a traditional culture-dish system but was limited in the amount of time tissue could be cultured. For decades the limiting factor in culturing intestinal cells was their life span. In the last decade, Dr. Hans Clever's lab developed a method for isolating intestinal crypts from the small or large intestine of mice and propagating LGR5^+^ stem cells from mice that can be continuously passaged and propagated (Sato et al., 2009), termed intestinal organoids. This scientific breakthrough has led to an exponential increase in publications using these models and opened an entire new world not previously and readily available. Additionally, in 2014 Yin et al., published a reproducible method to direct the differentiation of LGR5^+^ stem cells to become a specific cell type (i.e., enterocytes, goblet cells, stem cells, enteroendocrine cells; Yin et al., 2014). Directed differentiation methods have allowed researchers to examine cell-type specific responses and properties including barrier function and would be useful tools to examine host-microbe interactions (Pearce et al., 2018). Organoids can now be isolated from several species and methods for culturing stem cells from human biopsy tissue, termed human intestinal enteroids have been greatly refined. As organoids/enteroids have a variety of functional enteroendocrine cells, they are now thought to be a useful model to study the gut-brain axis (Hampton, 2017) which has potential implications for host-microbe interaction research. On the microbial side, human enteroids have been utilized for host-pathogen studies including *Enterohemorrhagic E. coli* (In et al., 2016), *Enterotoxogenic E. Coli* (Rajan et al., 2018), *Enteroaggretative E. Coli* (Noel et al., 2017), as well as Cholera toxin (Zomer-van Ommen et al., 2016). Additionally, host-commensal bacterial interactions including *Lactobacillus rhamnosus GG* (Aoki-Yoshida et al., 2016)*, L. acidophilus and Bacteroides thetaiotaomicron* have been examined. A full list of organisms, bacterial ligands, and bacterial metabolites utilizing, organoids and enteroids is listed in Table 1 along with main findings of each study. Examples of organoid morphology and structure of human and mouse samples are shown in Figure 1.

**Table 1:** Bacterial organisms and components that have been utilized for intestinal organoid research and main findings.

	**Organoid**	**Main Findings**	**References**
**PATHOGENIC**			
*Clostridium difficile*	iPS organoid	*C. difficile* inhibits Na^+^/H^+^ transport to create more favorable environment for growth. *C. difficile* decreases paracellular barrier function.	Engevik et al., 2015; Leslie et al., 2015
*Salmonella enterica*	iPS organoid, mouse ileal enteroid	*Salmonella* decreases stem cell markers and disrupts barrier function. *Salmonella* invades the epithelial barrier using vacuoles.	Zhang et al., 2014; Forbester et al., 2015
Enterohemorrhagic *E. coli*	Human colonoid, ileal/rectal enteroid	EHEC reduces mucus and affects the brush border cytoskeleton.	In et al., 2016
*E. coli* ECOR2	iPS organoid, duodenal enteroid	Colonization of organoids with non-pathogenic bacteria induces functional maturation of the intestinal barrier.	Hill et al., 2017
Enteroaggretative *E. coli*	Human duodenal/ileal enteroid, colonoid	EAEC adheres to small intestinal segments differently and is host specific.	Rajan et al., 2018
Enterotoxigenic *E. coli*	Human enteroid	Immune and epithelial cells coordinate to combat infection by improving barrier function and cytokine response.	Noel et al., 2017
*E. coli* O157:H7	iPS organoid	*E. coli* 0157:H7 causes loss of actin and epithelial integrity and increases ROS production and inflammatory cytokines.	Karve et al., 2017
*Helicobacter pylori*	iPS/human gastric organoid	Organoids are a good model to study *H. pylori* pathogenesis.	Pompaiah and Bartfeld, 2017
*Listeria monocytogenes*	Murine small intestinal enteroid	*L. monocytogenes* was used in enteroid protocol development for host-pathogen interactions.	Rothschild et al., 2016
Cholera toxin	Human jejunal/rectal enteroid	Enteroids were utilized to test preclinical drug inhibitors of Cholera toxin.	Zomer-van Ommen et al., 2016
**COMMENSAL**			
*Akkermansia muciniphila*	Murine ileal enteroid	*A. Muciniphila* modulates expression of important regulators of transcription factor regulation, cell cycle control, lipolysis, and satiety.	Lukovac et al., 2014
*Faecalibacterium prausnitzii*	Murine ileal enteroid	*F. prausnitzii* weakly promotes host regulation of cellular processes, neurological system processes, and cell differentiation.	Lukovac et al., 2014
*Lactobacillus acidophilus*	Chicken enteroid	*L. acidophilus* promotes organoid growth and increases production of beneficial prostaglandins.	Pierzchalska et al., 2017
*Lactobacillus rhamnosus GG*	Murine small intestine enteroid	*L. rhamnosus* GG increases toll-like receptor 3 expression.	Aoki-Yoshida et al., 2016
*Bacteroides thetaiotaomicron*	Murine ileal enteroid	*B. thetaiotaomicron* induces host epithelial changes via increased fucosylation	Engevik et al., 2013
**BACTERIAL LIGANDS**			
Lipopolysaccharide	Murine colonoid	LPS is the dominant bacterial agonist that affects cell proliferation in a toll-like receptor 4 dependent manner.	Naito et al., 2017
Flagellin	Murine small intestinal enteroid	Flagellin was used in protocol development to study host-microbe interactions.	Rothschild et al., 2016
Poly(I:C)	Murine small intestine enteroid	Poly(I:C) combined with *L. rhamnosus* GG increases expression of interferon-α and chemokine CXCL1	Aoki-Yoshida et al., 2016
Muramyl-dipeptide	Murine small intestinal enteroid	MDP induces higher yield of intestinal organoids, and induces stem cell protection via NOD2.	Nigro et al., 2014
**BACTERIAL METABOLITES**			
Short-chain fatty acids	Murine small intestinal crypts	Propionate and butyrate but not acetate regulation expression of HDAC activity, and expression of adipose related genes Fiaf, PPARγ and Gpr3. SCFA's promote organoid development. Butyrate stimulates epithelial production of retinoic acid via HDAC inhibition.	Lukovac et al., 2014; Park et al., 2016; Schilderink et al., 2016
Indoleacrylic Acid	Human colonoid	IA promotes intestinal epithelial barrier function and reduces inflammation.	Wlodarska et al., 2017

Another source of primary human intestinal epithelium are derived from inducible pluripotent stem cells (iPSC), also known as human intestinal organoids (HIO). These organoids are generated from iPSC's by a multistep process first involving development of an endoderm, and later development of epithelial and organoid structures (Workman et al., 2018). These iPS organoids have been utilized to study how *clostridium difficile* infection affects ion transport (Engevik et al., 2015) as well as intestinal barrier function (Leslie et al., 2015). Additionally they have been used to study bacterial colonization of *E. coli ECOR2* effects on the host (Hill et al., 2017), host response to infection of Shiga toxin producing *E. coli*, (Karve et al., 2017) and *Helicobacter pylori* pathogenesis in gastric organoids (Pompaiah and Bartfeld, 2017).

In 3D culture, microbiological research of organoids and enteroids is highly difficult. The 3D architecture of these cell systems causes their polarity to be “inside-out” where the lumen is contained in the center of the spherical structure. Accessing the lumen inside the organoid, where bacteria normally reside, is a significant challenge that has been overcome by various laboratories (Hill et al., 2017). Several researchers have found ways to by-pass this via microinjection or trituration (Karve et al., 2017); however, this is a very difficult and time consuming process. In the last few years, there have been large steps forward in organoid technology including the ability to polarize these cells on a 2D monolayer as well as the ability to co-culture with immune cells, neurons and other cell types to make a more physiologically relevant GI model system.

#### Organs-on-a-chip and microfluidic devices

Organs on a chip utilizing microfluidics is the most recent bioengineering advancement as it pertains to intestinal cell models and may show the greatest potential to mimic complex multi-organ or multi-layer systems found *in vivo*. A recent comprehensive review on organ-on-a-chip models was written by Bein et al. (Kasendra et al., 2018). Several variations of these models now exist and the field is relatively new but expanding at a very rapid rate. One example of the applications for this type of platform utilizes Caco-2 cells cultured in the presence of planar stretch and luminal flow that mimics digestive shear forces and presumably promotes a more physiologically-relevant state of the cells. Researchers have used this model to screen several different applications including evaluating anti-inflammatory probiotics and analyzing bacterial overgrowth often observed with IBD (Kim et al., 2016b). Additionally, gut-on-a-chip microfluidic devices allow for co-culturing of living microbiome and engineered human intestines (Kim et al., 2016a). Recent advancements in gut-on-chip technology has allowed for electrodes to be embedded into the chips in order to analyze transepithelial electrical resistance (TER) a common functional measure of intestinal barrier integrity (Henry et al., 2017). More recently, a small intestine-on-a-chip has been created using human biopsy derived organoids cultured alone or in conjunction with intestinal microvascular endothelial cells to better mimic the complexity of the *in vivo* intestinal epithelium tissue (Kasendra et al., 2018). A recent more comprehensive review on these models was recently published (Kasendra et al., 2018).

## Advantages and disadvantages of *ex vivo* models

Condensed advantages/disadvantages of both *in vitro* and *ex vivo* models are shown in Table 2. Overall, *ex vivo* systems contain added complexity and functional cross-talk between many different cell types that are not generally found in *in vitro systems* (Roeselers et al., 2013). The IntesTINE system has potential to be a very useful model to study bacterial-host interactions in live tissue of a physiologically relevant animal model. However, it has not been fully validated for use and therefore may provide some challenges. Ussing chambers can utilize live mammalian tissue or cultured cells on snap-well dishes. Tissues or cells can be treated before or during Ussing chamber runs and more than one analysis can be conducted including epithelial resistance, FITC-LPS, or FITC-Dextran transport, as well as measures of glucose or amino acid transport. The main disadvantage to this system is that the tissue is only viable for a period of hours and it cannot be used for longer term studies (>5 h). Intestinal organoids, especially from human biopsy samples provide a near-ideal model to utilize for host-microbe interactions. Cell types found in organoids recapitulate the normal epithelium and translatability if using human organoids is not an issue. For bacterial research, the main drawback is the 3D organoid system that has the lumen internalized and provides difficulty in treating with bacteria; however, recent host-pathogen studies have emerged that utilize intestinal organoids grown as confluent monolayers. Organs-on-a-chip are technically challenging and working with small volumes and cell numbers can limit the methods that can be used for downstream analysis. Many times end-point studies alone are used because monitoring changes overtime in a chip is challenging and many methods of detecting cellular changes involve terminating the cells. However, the industry involvement in organ-on-a-chip technology (ALine, Draper) has improved reproducibility and manufacture of chips built to a researchers specifications. Another major disadvantage to the current *ex vivo* systems is the inability of these systems to recapitulate the anaerobic environment of the intestinal lumen as well as the oxygen gradient established at the mucosal layer. With the growing field, increased interest, available organ types and advances in real-time monitoring, it may be possible to replace rodent or other animal models in a variety of scientific applications in the near future. Condensed advantages/disadvantages are shown in Table 2.

**Table 2:** Advantages and Disadvantages of intestinal host models.

	**Advantages**	**Disadvantages**
***IN VITRO***		
Caco-2	Can be polarized, cost effective, ease of use, extensive literature available, commercially available	Cancerous origin, difficult to control differentiation
HT-29	Can be polarized, commercially available, mucus producing cells	Cancerous origin
T84	Can be polarized, commercially available, cost effective, ease of use	Cancerous origin
IEC-6	Can be polarized, commercially available, non-cancerous origin, good for studying small intestine	Not good mimic for colon
IPEC-J2	Can be polarized, contain multiple cell types, commercially available, good for studying small intestine, non-cancerous origin, physiologic relevance to humans	Not good mimic for colon
***EX VIVO***		
Ussing Chamber	Tissue is polarized, can obtain barrier function and transport data, contain all epithelial cell types	Short Term (up to 5 h)
Organoids	Can be grown 2 or 3D, can be polarized, contain all epithelial cell types, contain transporters and pattern recognition receptors	Human samples difficult to obtain, cost
Gut-on-Chip	More complex system, more physiologically relevant	Cost, not commercially available
**OTHER**		
Co-culture	More complex system, cell type interactions, more physiologically relevant	Difficult to control conditions for multiple cell types
Tissue Engineering	Translatability	Cost, not fully validated

## Other potentially useful models

### Co-culture

With the advancements in technology, including organs-on-a-chip, have come a new wave of more complex, integrated cell systems including the gut-brain axis and immune systems. Advances have been made using Transwell culture plates, where cells and bacteria can be co-cultured and separated by a semi-permeable membrane that allow for bacteria-host interactions to be investigated in a complex culture. For example, Caco-2 cells were grown in a Transwell® system where epithelial cells are grown in the apical chamber, while human dendritic cells are cultured in the basolateral chamber, followed by apical exposure to live probiotic *Lactobacillus paracasei*. This co-culture model showed that probiotics in the presence of epithelial and immune cells exert a different response than observed using single cell cultures (Bermudez-Brito et al., 2015). Additionally, flow and variation in the environment between a bacteria and host layers can be applied, with the added advantage of the larger size then organ-on-a-chip models. Human non-transformed neonatal small intestinal cells (H4-1) have been used to study bacterial interactions when co-cultured with macrophages and researchers observed a significant difference in bacterial translocation in epithelial cells cultured alone vs. those co-cultured with immune cells (Trapecar et al., 2014). Additionally, a recent model was developed that contains macrophages co-cultured with human organoids (Noel et al., 2017). Another microfluidics system is the HuMiX which incorporates human cells co-cultured with bacterial cells (Shah et al., 2016). Advantages here include more complex systems that may more accurately recapitulate *in vivo* physiology but disadvantages include commercial availability, cost, and culturing conditions.

### Tissue engineering

A comprehensive review of tissue engineering of the gut was written by Bitar and Raghavan in 2012 (Bitar et al., 2014). In brief, tissue engineering involves generation, or in many cases regeneration of complex tissue systems. For the intestine, this includes tubular tissue constructs that contain absorptive villi and crypts, along with associated smooth muscle and enteric nerves to provide a fully integrated system. Cell sources for this can be difficult but strides have been made utilizing stem cells to direct differentiation to a desired cell type.

### Host-microbial metabolite models

Dietary tryptophan that is metabolized by both host and bacteria to indole derivatives have been shown to reduce intestinal permeability in T84 cells (Jennis et al., 2018). Intestinal organoids have proven to be a good model to examine mechanisms of host-bacterial interactions in a multi-cellular system (Rothschild et al., 2016; Blutt et al., 2018). Polyphenol metabolites which are biotransformed by the gut microbiota, have also been examined *in vitro* models (mainly Caco-2) including urolithin A (Gonzalez-Sarrias et al., 2015) and tert-butyl hydroperoxide (Deiana et al., 2010) and 3,4-dihydroxyphenyl-ethanol (Manna et al., 1997). Metabolic products produced by bacteria, especially butyrate, have been implicated in intestinal health and Caco-2 cells have been utilized in several studies to elucidate the mechanisms by which SCFA's modulate colonic function (Nepelska et al., 2012).

## *In vitro* and *ex vivo* models for military relevant stressor research

As mentioned earlier, there are several environmental and physical stressors that are experienced by human beings, and in the military these stressors are often exacerbated. Several of these stressor types can be mimicked in the aforementioned *in vivo* and *ex vivo* models to provide a more mechanistic approach that can be utilized for future clinical research. Currently no data is available pertaining to model systems for military specific research in this area.

### Heat stress/cold stress

Heat stress (HS) has been studied *in vitro* and *ex vivo* in a number of models covered in this review. Generally, in *in vitro* models, heat stress/hyperthermia is applied using a temperature adjustment on a standard cell culture incubator while in many *ex vivo* models the stressor is applied to the animal prior to tissue being excised, such as in Ussing Chambers. Caco-2 cells have been used to study heat stress effects on the gut (Swank et al., 1998; Hershko et al., 2003; Dokladny et al., 2006; Xiao et al., 2013) to show changes in intestinal permeability, heat shock response, and immune response that mimic human *in vivo* studies. Similarly, IEC-6 cells have been utilized to study heat stress mechanisms and cell death (Xu et al., 1996; Yu et al., 2013). IPEC-J2 cells have been utilized to show that HS affects tight junctions, as well as selenoproteins involved in the oxidative response. In *ex vivo* models, heat stress has been studied extensively using post-stress measurement of barrier function in Ussing chambers (Pearce et al., 2013, 2014). Cold stress has not been studied *in vitro*, and to date there are no *in vitro* models of environmental cold stress.

### High altitude/hypoxia

Hypoxia, mimicking high altitude has been studied extensively in *in vitro* models, including Caco-2 (Unno et al., 1996; Xu et al., 1999; Lee et al., 2002; Lei et al., 2014; Jin and Blikslager, 2016), IEC-6 (Xu et al., 1999; Li et al., 2003; Miki et al., 2004; Chen et al., 2010) as well as IPEC-J2. This can be a chemically induced hypoxia using a hypoxia-mimetic agent Cobalt Chloride, or environmentally induced utilizing *in vitro* hypoxia chambers that can control oxygen and carbon dioxide concentrations. These studies have mechanistically shown the involvement of myosin light chain kinase in hypoxia-induced barrier dysfunction (Jin and Blikslager, 2016). Interestingly, one study in Caco-2 cells shows that exposing host cells to hypoxia prior to bacteria treatment decreased bacterial internalization of *Yersinia enterocolitica* (Zeitouni et al., 2016). Hypoxia has also been studied in colonic biopsies in Ussing chambers where hypoxia is induced in one or both sides of the chamber (Carra et al., 2013). Additionally, hypoxia combined with *E. coli* exacerbates intestinal inflammation and cytokine secretion in a Ussing chamber hypoxic model (Ding et al., 2001). Additionally, hypobaric hypoxia, which more accurately mimics environmental hypoxia, such as is seen at high altitudes, has been shown to alter intestinal barrier function in an *ex vivo* rodent model (Saravi et al., 2002)

### Nutritional stress

Nutrient deprivation, including amino acid deprivation (Roussel et al., 2017), folate deprivation (Townsend et al., 2004), fasting (Le Bacquer et al., 2003) have been examined in Caco-2 cells, while glucose deprivation has been studied in depth utilizing HT-29 cells (Hwang et al., 2008; Li et al., 2009). Intestinal organoids are starting to be utilized for more nutritional related research and effects of essential amino acid deprivation have been studied in intestinal stem cells (Saito et al., 2017).

## “OMICS” and bioinformatics methods for *in vitro* and *ex vivo* models

To determine effects of microbes or stressors on the host, or bacteria there are a number of functional, quantitative, and qualitative measurements that have been standard practice. However, in recent years, “Omics” techniques that have been applied to human and animal models have come to the forefront of analyses. Valuable information can obtained through the use of “omics” approaches in host-microbe or host-metabolite models as they integrate mammalian cells and microbial cells or microbial by-products. Experimental information about the function via RNA (transcriptomics), protein analysis (proteomics), proteins that are externally secreted by cells (secretomes) (Mukherjee and Mani, 2013) and the ability to identify the presence of metabolites (metabolomics) are potential targets. Also, the microbial diversity of a specific community and its genes could be extensively analyzed from any of these models with the current Next-generation sequencing techniques using specific markers, such as the 16s rRNA gene (metagenomics). To date, with advances in bioinformatics, researchers have access to a variety of computational methods to analyze “omics” data (Segata et al., 2013) and the combination of different approaches like metagenomics and metaproteomics which not only reveal the taxonomy, but also functional activity.

There are several RNA-Bioinformatics tools comprised in different packages, like RNA workbench (Gruning et al., 2017), for the analysis of RNA structures, RNA alignments, RNA-RNA interactions, RNA-protein interactions, RNA sequencing, ribosome profiling, and genome annotation. An important resource since 1995 is the Kyoto Encyclopedia of Genes and Genomes (KEGG) integrated database that can bring information about metabolism and cellular processes from high-throughput genome sequence data (Kanehisa et al., 2014). Another tool for the study of metabolic pathways and enzymes is the MetaCyc reference database (Caspi et al., 2008).

Information garnered from “omics” techniques may set a basis to employ a helpful approach called machine learning. It consists of a series of algorithms that after “trained,” can predict outcomes and future states in specific areas of the research field, such as shifts in the microbiome structure and function as a result of certain factors (e.g., health vs. disease status; Yazdani et al., 2016). One common machine learning technique is the random forest regression [reviewed on (Knights et al., 2011; Knight et al., 2018)] that generates a large number of trees to select for the best one to carry out taxonomy classification, referring specially to microbiome studies (Breiman, 2001). The integration of these methods could provide information about patterns and understanding on microorganism's abundance as a consequence of a stressor.

## Conclusions, future plans and implications for military research

**Figure 2:**
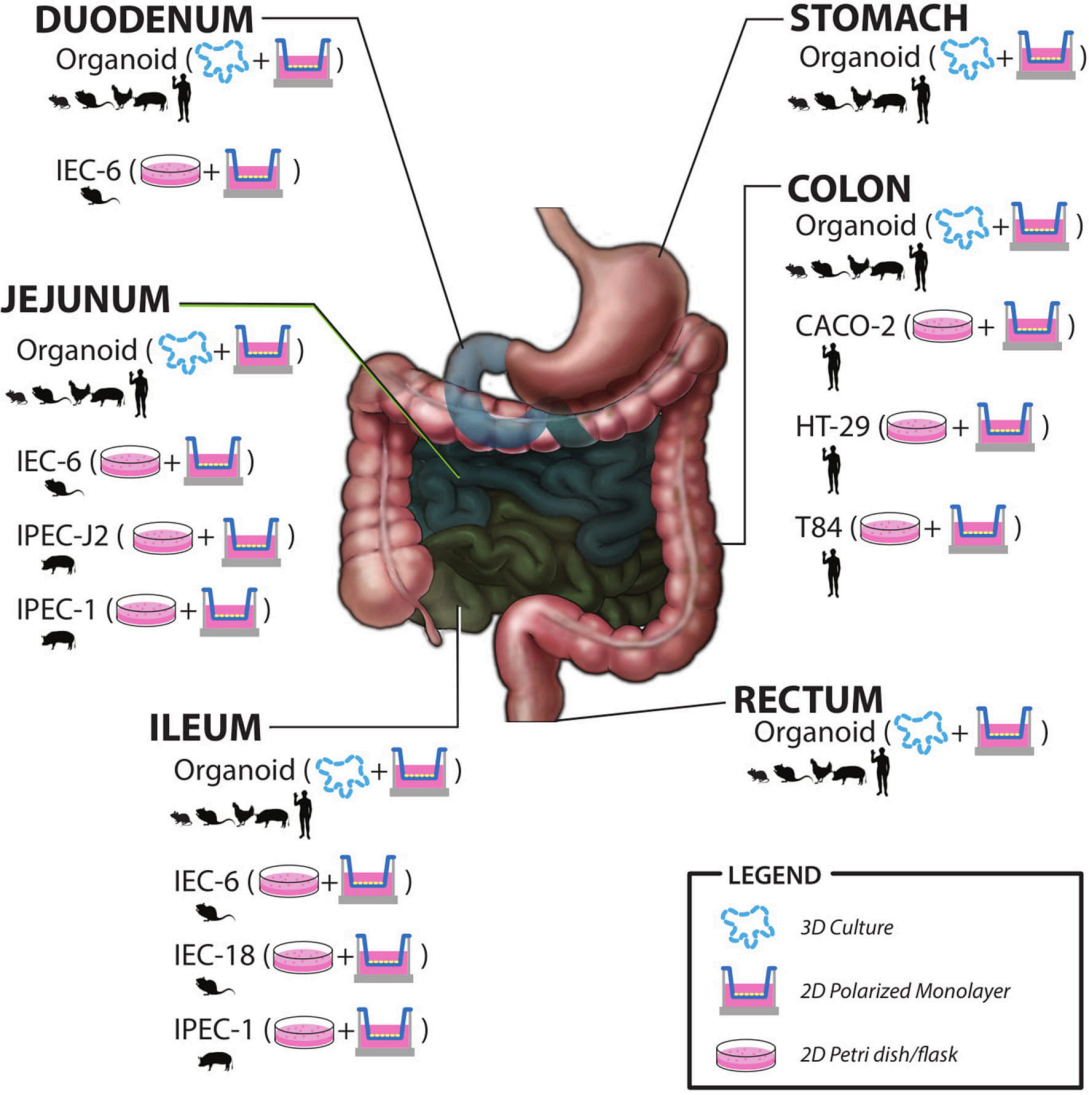
Summary of *in vitro* and *ex vivo* models including species origin, intestinal region, and most commonly utilized culture methods.

As the microbiome field advances it is becoming clearer that humans rely heavily on their microbial counterparts to maintain intestinal homeostasis. The gut microbiota can respond, in parallel with the host to changes in environment, diet, and other common types of stress and this can lead to dysbiosis. This is often compounded in members of the military who often experience stressors in combination during training or on the battlefield. Although there are emerging studies of these stressors in human models, there is a need to further characterize the effects of these stressors via human studies using the military cohort. There is also a need to understand the mechanisms of action via basic and early applied research in physiologically relevant *in vitro* and *ex vivo* models. There have been *in vitro* host models utilized to study host-microbe interactions for the better part of 50 years. New advances in cell biology have allowed for host models much more similar to a healthy human host. The ability to study physiologically relevant models, including the ability to examine specific cell types as well as differentiated vs. undifferentiated allows for in depth examination of cell-specific responses. This is especially true for crypt-residing stem and Paneth cells that will experience microbial and microbial metabolite exposure during times of injury, stress, or dysbiosis. Future research to study host-microbiome interactions using primary cell culture models, such as intestinal organoids will provide new insights into the host-microbe cross-talk. Human gut-on-a-chip platforms also provide a great deal of innovation and opportunity to study the interactions on a more physiologically relevant level. These models can be individualized and used to study military-relevant stressor effects between the gut microbiota and human host and provide input to more applied clinical research studies. There is also potential research areas using new *ex vivo* models to examine the effects of bacterial metabolites on host function. A graphic summary of models discussed in this review is shown in Figure 2.

Additionally, clinical studies can help inform the *in vitro/ex vivo* work to explore potential mechanisms. One example might be that a military-relevant stressor, such as high altitude or nutrient deprivation has been identified. This can be modeled in host models to determine effects on the host, as well as in *in vitro* fermentation models to examine effects on bacterial function and metabolites. Thereafter, a potential mitigation strategy can be introduced into both systems and depending on the outcomes, can be applied to clinical research. Ultimately, the goal is to provide potential mitigation strategies to improve the health and performance of US warfighters.

## Author contributions

SP designed, wrote, and edited the paper. KR helped design and edit the paper. HC, JK, IP-F and NZ helped write and edit the paper.

### Conflict of interest statement

The authors declare that the research was conducted in the absence of any commercial or financial relationships that could be construed as a potential conflict of interest.

## References

[B1] Aleksandrova K. Romero-Mosquera B. Hernandez V. (2017). Diet, gut microbiome and epigenetics: emerging links with inflammatory bowel diseases and prospects for management and prevention. Nutrients 9:E962. 10.3390/nu9090962 28867793 PMC5622722

[B2] Alexander D. A. Klein S. (2009). First responders after disasters: a review of stress reactions, at-risk, vulnerability, and resilience factors. Prehosp. Disaster Med. 24, 87–94. 10.1017/S1049023X00006610 19591301

[B3] Allen J. M. Mailing L. J. Cohrs J. Salmonson C. Fryer J. D. Nehra V.. (2017). Exercise training-induced modification of the gut microbiota persists after microbiota colonization and attenuates the response to chemically-induced colitis in gnotobiotic mice. Gut Microbes 9, 115–130. 10.1080/19490976.2017.1372077 28862530 PMC5989796

[B4] Altamimi M. Abdelhay O. Rastall R. A. (2016). Effect of oligosaccharides on the adhesion of gut bacteria to human HT-29 cells. Anaerobe 39, 136–142. 10.1016/j.anaerobe.2016.03.010 27018325

[B5] Anand A. C. Sashindran V. K. Mohan L. (2006). Gastrointestinal problems at high altitude. Trop. Gastroenterol. 27, 147–153. 17542291

[B6] Aoki-Yoshida A. Saito S. Fukiya S. Aoki R. Takayama Y. Suzuki C.. (2016). *Lactobacillus rhamnosus* GG increases toll-like receptor 3 gene expression in murine small intestine *ex vivo* and *in vivo*. Benef. Microbes 7, 421–429. 10.3920/BM2015.0169 27013459

[B7] Arboleya S. Bahrami B. Macfarlane S. Gueimonde M. Macfarlane G. T. de los Reyes-Gavilan C. G. (2015). Production of immune response mediators by HT-29 intestinal cell-lines in the presence of bifidobacterium-treated infant microbiota. Benef. Microbes 6, 543–552. 10.3920/BM2014.0111 25691102

[B8] Arce C. Ramirez-Boo M. Lucena C. Garrido J. J. (2010). Innate immune activation of swine intestinal epithelial cell lines (IPEC-J2 and IPI-2I) in response to LPS from *Salmonella typhimurium*. Comp. Immunol. Microbiol. Infect. Dis. 33, 161–174. 10.1016/j.cimid.2008.08.003 18799216

[B9] Beltinger J. del Buono J. Skelly M. M. Thornley J. Spiller R. C. Stack W. A.. (2008). Disruption of colonic barrier function and induction of mediator release by strains of *Campylobacter jejuni* that invade epithelial cells. World J. Gastroenterol. 14, 7345–7352. 10.3748/wjg.14.7345 19109868 PMC2778118

[B10] Bermudez-Brito M. Munoz-Quezada S. Gomez-Llorente C. Matencio E. Romero F. Gil A. (2015). *Lactobacillus paracasei* CNCM I-4034 and its culture supernatant modulate Salmonella-induced inflammation in a novel transwell co-culture of human intestinal-like dendritic and Caco-2 cells. BMC Microbiol. 15:79. 10.1186/s12866-015-0408-6 25887178 PMC5353866

[B11] Bitar K. N. Raghavan S. Zakhem E. (2014). Tissue engineering in the gut: developments in neuromusculature. Gastroenterology 146, 1614–1624. 10.1053/j.gastro.2014.03.044 24681129 PMC4035447

[B12] Blutt S. E. Crawford S. E. Ramani S. Zou W. Y. Estes M. K. (2018). Engineered human gastrointestinal cultures to study the microbiome and infectious diseases. Cell. Mol. Gastroenterol. Hepatol. 5, 241–251. 10.1016/j.jcmgh.2017.12.001 29675450 PMC5904028

[B13] Bonde J. P. Utzon-Frank N. Bertelsen M. Borritz M. Eller N. H. Nordentoft M.. (2016). Risk of depressive disorder following disasters and military deployment: systematic review with meta-analysis. Br. J. Psychiatry 208, 330–336. 10.1192/bjp.bp.114.157859 26892850

[B14] Botic T. Klingberg T. D. Weingartl H. Cencic A. (2007). A novel eukaryotic cell culture model to study antiviral activity of potential probiotic bacteria. Int. J. Food Microbiol. 115, 227–34. 10.1016/j.ijfoodmicro.2006.10.044 17261339

[B15] Boyen F. Pasmans F. Van Immerseel F. Donne E. Morgan E. Ducatelle R.. (2009). Porcine *in vitro* and *in vivo* models to assess the virulence of *Salmonella enterica* serovar *Typhimurium* for pigs. Lab. Anim. 43, 46–52. 10.1258/la.2007.007084 18987064

[B16] Breiman L. (2001). Random forests. Mach. Learn. 45, 5–32. 10.1023/A:1010933404324

[B17] Brosnahan A. J. Brown D. R. (2012). Porcine IPEC-J2 intestinal epithelial cells in microbiological investigations. Vet. Microbiol. 156, 229–237. 10.1016/j.vetmic.2011.10.017 22074860 PMC3289732

[B18] Browning T. H. Trier J. S. (1969). Organ culture of mucosal biopsies of human small intestine. J. Clin. Invest. 48, 1423–1432. 10.1172/JCI106108 5796354 PMC322369

[B19] Cani P. D. Geurts L. Matamoros S. Plovier H. Duparc T. (2014). Glucose metabolism: focus on gut microbiota, the endocannabinoid system and beyond. Diabetes Metab. 40, 246–257. 10.1016/j.diabet.2014.02.004 24631413

[B20] Carabotti M. Scirocco A. Maselli M. A. Severi C. (2015). The gut-brain axis: interactions between enteric microbiota, central and enteric nervous systems. Ann. Gastroenterol. 28, 203–209. 25830558 PMC4367209

[B21] Carra G. E. Ibanez J. E. Saravi F. D. (2013). The effect of acute hypoxia on short-circuit current and epithelial resistivity in biopsies from human colon. Digest. Dis. Sci. 58, 2499–2506. 10.1007/s10620-013-2711-0 23695875

[B22] Caspi R. Foerster H. Fulcher C. A. Kaipa P. Krummenacker M. Latendresse M.. (2008). The MetaCyc database of metabolic pathways and enzymes and the BioCyc collection of pathway/genome databases. Nucleic Acids Res. 36, D.623–D.631. 10.1093/nar/gkr1014 17965431 PMC2238876

[B23] Chen H. Q. Yang J. Zhang M. Zhou Y. K. Shen T. Y. Chu Z. X.. (2010). Lactobacillus plantarum ameliorates colonic epithelial barrier dysfunction by modulating the apical junctional complex and PepT1 in IL-10 knockout mice. Am. J. Physiol. Gastrointest. Liver Physiol. 299, G1287–G1297. 10.1152/ajpgi.00196.2010 20884889

[B24] Chevalier C. Stojanovic O. Colin D. J. Suarez-Zamorano N. Tarallo V. Veyrat-Durebex C.. (2015). Gut microbiota orchestrates energy homeostasis during cold. Cell 163, 1360–1374. 10.1016/j.cell.2015.11.004 26638070

[B25] Cinova J. De Palma G. Stepankova R. Kofronova O. Kverka M. Sanz Y.. (2011). Role of intestinal bacteria in gliadin-induced changes in intestinal mucosa: study in germ-free rats. PLoS ONE 6:e16169. 10.1371/journal.pone.0016169 21249146 PMC3020961

[B26] Clark A. Mach N. (2016). Exercise-induced stress behavior, gut-microbiota-brain axis and diet: a systematic review for athletes. J. Int. Soc. Sports Nutr. 13:43. 10.1186/s12970-016-0155-6 27924137 PMC5121944

[B27] Clarke G. Stilling R. M. Kennedy P. J. Stanton C. Cryan J. F. Dinan T. G. (2014). Minireview: gut microbiota: the neglected endocrine organ. Mol. Endocrinol. 28, 1221–1238. 10.1210/me.2014-1108 24892638 PMC5414803

[B28] Coskun T. Yegen B. C. Alican I. Peker O. Kurtel H. (1996). Cold restraint stress-induced gastric mucosal dysfunction. Role of nitric oxide. Digest. Dis. Sci. 41, 956–963. 10.1007/BF02091537 8625769

[B29] Creamer M. Wade D. Fletcher S. Forbes D. (2011). PTSD among military personnel. Int. Rev. Psychiatry 23, 160–165. 10.3109/09540261.2011.559456 21521085

[B30] Dahiya R. Lesuffleur T. Kwak K. S. Byrd J. C. Barbat A. Zweibaum A.. (1992). Expression and characterization of mucins associated with the resistance to methotrexate of human colonic adenocarcinoma cell line HT29. Cancer Res. 52, 4655–4662. 1511431

[B31] David L. A. Weil A. Ryan E. T. Calderwood S. B. Harris J. B. Chowdhury F.. (2015). Gut microbial succession follows acute secretory diarrhea in humans. MBio 6:e00381-15. 10.1128/mBio.00381-15 25991682 PMC4442136

[B32] De Simone V. Franze E. Ronchetti G. Colantoni A. Fantini M. C. Di Fusco D.. (2015). Th17-type cytokines, IL-6 and TNF-alpha synergistically activate STAT3 and NF-kB to promote colorectal cancer cell growth. Oncogene 34, 3493–3503. 10.1038/onc.2014.286 25174402 PMC4493653

[B33] Deiana M. Corona G. Incani A. Loru D. Rosa A. Atzeri A.. (2010). Protective effect of simple phenols from extravirgin olive oil against lipid peroxidation in intestinal Caco-2 cells. Food Chem. Toxicol. 48, 3008–3016. 10.1016/j.fct.2010.07.041 20691238

[B34] Deitch E. A. Haskel Y. Cruz N. Xu D. Kvietys P. R. (1995). Caco-2 and IEC-18 intestinal epithelial cells exert bactericidal activity through an oxidant-dependent pathway. Shock 4, 345–350. 10.1097/00024382-199511000-00006 8595521

[B35] Ding J. Magnotti L. J. Huang Q. Xu D. Z. Condon M. R. Deitch E. A. (2001). Hypoxia combined with *Escherichia coli* produces irreversible gut mucosal injury characterized by increased intestinal cytokine production and DNA degradation. Shock 16, 189–195. 10.1097/00024382-200116030-00004 11531020

[B36] Doe W. F. (1989). The intestinal immune system. Gut 30, 1679–1685. 10.1136/gut.30.12.1679 2693229 PMC1434429

[B37] Dokladny K. Moseley P. L. Ma T. Y. (2006). Physiologically relevant increase in temperature causes an increase in intestinal epithelial tight junction permeability. Am. J. Physiol. Gastrointest. Liver Physiol. 290, G204–G212. 10.1152/ajpgi.00401.2005 16407590

[B38] Dokladny K. Zuhl M. N. Moseley P. L. (2016). Intestinal epithelial barrier function and tight junction proteins with heat and exercise. J. Appl. Physiol. (1985) 120, 692–701. 10.1152/japplphysiol.00536.2015 26359485 PMC4868372

[B39] Donowitz M. Keusch G. T. Binder H. J. (1975). Effect of Shigella enterotoxin on electrolyte transport in rabbit ileum. Gastroenterology 69, 1230–1237. 172398

[B40] Eckburg P. B. Bik E. M. Bernstein C. N. Purdom E. Dethlefsen L. Sargent M.. (2005). Diversity of the human intestinal microbial flora. Science 308, 1635–1638. 10.1126/science.1110591 15831718 PMC1395357

[B41] Elson C. O. Alexander K. L. (2015). Host-microbiota interactions in the intestine. Dig. Dis. 33, 131–136. 10.1159/000369534 25925913

[B42] Engevik M. A. Aihara E. Montrose M. H. Shull G. E. Hassett D. J. Worrell R. T. (2013). Loss of NHE3 alters gut microbiota composition and influences Bacteroides thetaiotaomicron growth. Am. J. Physiol. Gastrointest. Liver Physiol. 305, G697–G711. 10.1152/ajpgi.00184.2013 24072680 PMC3840232

[B43] Engevik M. A. Engevik K. A. Yacyshyn M. B. Wang J. Hassett D. J. Darien B.. (2015). Human clostridium difficile infection: inhibition of NHE3 and microbiota profile. Am. J. Physiol. Gastrointest. Liver Physiol. 308, G497–G509. 10.1152/ajpgi.00090.2014 25552580 PMC4422371

[B44] Everson C. A. Toth L. A. (2000). Systemic bacterial invasion induced by sleep deprivation. Am. J. Physiol. Regul. Integr. Comp. Physiol. 278, R905–R916. 10.1152/ajpregu.2000.278.4.R905 10749778

[B45] Farzi A. Frohlich E. E. Holzer P. (2018). Gut microbiota and the neuroendocrine system. Neurotherapeutics 15, 5–22. 10.1007/s13311-017-0600-5 29380303 PMC5794709

[B46] Forbester J. L. Goulding D. Vallier L. Hannan N. Hale C. Pickard D.. (2015). Interaction of *Salmonella enterica* serovar *typhimurium* with intestinal organoids derived from human induced pluripotent stem cells. Infect. Immun. 83, 2926–2934. 10.1128/IAI.00161-15 25964470 PMC4468523

[B47] Frasca L. Lande R. (2012). Role of defensins and cathelicidin LL37 in auto-immune and auto-inflammatory diseases. Curr. Pharm. Biotechnol. 13, 1882–1897. 10.2174/138920112802273155 22250708

[B48] Friedl K. E. Moore R. J. Martinez-Lopez L. E. Vogel J. A. Askew E. W. Marchitelli L. J.. (1994). Lower limit of body fat in healthy active men. J. Appl. Physiol. (1985) 77, 933–940. 10.1152/jappl.1994.77.2.933 8002550

[B49] Furrie E. Macfarlane S. Thomson G. Macfarlane G. T. (2005). Toll-like receptors-2,−3 and−4 expression patterns on human colon and their regulation by mucosal-associated bacteria. Immunology 115, 565–574. 10.1111/j.1365-2567.2005.02200.x 16011525 PMC1782176

[B50] Galley J. D. Bailey M. T. (2014). Impact of stressor exposure on the interplay between commensal microbiota and host inflammation. Gut Microbes 5, 390–396. 10.4161/gmic.28683 24690880 PMC4153778

[B51] Gonzalez-Sarrias A. Tome-Carneiro J. Bellesia A. Tomas-Barberan F. A. Espin J. C. (2015). The ellagic acid-derived gut microbiota metabolite, urolithin A, potentiates the anticancer effects of 5-fluorouracil chemotherapy on human colon cancer cells. Food Funct. 6, 1460–1469. 10.1039/C5FO00120J 25857357

[B52] Gruning B. A. Fallmann J. Yusuf D. Will S. Erxleben A. Eggenhofer F.. (2017). The RNA workbench: best practices for RNA and high-throughput sequencing bioinformatics in galaxy. Nucleic Acids Res. 45, W560–W566. 10.1093/nar/gkx409 28582575 PMC5570170

[B53] Guarino A. Cohen M. Thompson M. Dharmsathaphorn K. Giannella R. (1987). T84 cell receptor binding and guanyl cyclase activation by Escherichia coli heat-stable toxin. Am. J. Physiol. 253, G775–G780. 10.1152/ajpgi.1987.253.6.G775 2892417

[B54] Hampton T. (2017). Organoids reveal clues to gut-brain communication. JAMA 318, 787–788. 10.1001/jama.2017.11545 28873141

[B55] Hecht G. Marrero J. A. Danilkovich A. Matkowskyj K. A. Savkovic S. D. Koutsouris A.. (1999). Pathogenic *Escherichia coli* increase Cl-secretion from intestinal epithelia by upregulating galanin-1 receptor expression. J. Clin. Invest. 104, 253–262. 10.1172/JCI6373 10430606 PMC408417

[B56] Henning P. C. Park B. S. Kim J. S. (2011). Physiological decrements during sustained military operational stress. Mil. Med. 176, 991–997. 10.7205/MILMED-D-11-00053 21987955

[B57] Henry O. Y. F. Villenave R. Cronce M. J. Leineweber W. D. Benz M. A. Ingber D. E. (2017). Organs-on-chips with integrated electrodes for trans-epithelial electrical resistance (TEER) measurements of human epithelial barrier function. Lab. Chip 17, 2264–2271. 10.1039/C7LC00155J 28598479 PMC5526048

[B58] Hering N. A. Luettig J. Krug S. M. Wiegand S. Gross G. van Tol E. A.. (2017). Lactoferrin protects against intestinal inflammation and bacteria-induced barrier dysfunction *in vitro*. Ann. N. Y. Acad. Sci. 1405, 177–188. 10.1111/nyas.13405 28614589

[B59] Hershko D. D. Robb B. W. Luo G. J. Paxton J. H. Hasselgren P. O. (2003). Interleukin-6 induces thermotolerance in cultured Caco-2 cells independent of the heat shock response. Cytokine 21, 1–9. 10.1016/S1043-4666(02)00488-X 12668153

[B60] Hill D. R. Huang S. Nagy M. S. Yadagiri V. K. Fields C. Mukherjee D.. (2017). Bacterial colonization stimulates a complex physiological response in the immature human intestinal epithelium. Elife 6:e29132. 10.7554/eLife.29132 29110754 PMC5711377

[B61] Holmes E. Li J. V. Marchesi J. R. Nicholson J. K. (2012). Gut microbiota composition and activity in relation to host metabolic phenotype and disease risk. Cell Metab. 16, 559–64. 10.1016/j.cmet.2012.10.007 23140640

[B62] Hooper L. V. Littman D. R. Macpherson A. J. (2012). Interactions between the microbiota and the immune system. Science 336, 1268–73. 10.1126/science.1223490 22674334 PMC4420145

[B63] Huet G. Kim I. de Bolos C. Lo-Guidice J. M. Moreau O. Hemon B.. (1995). Characterization of mucins and proteoglycans synthesized by a mucin-secreting HT-29 cell subpopulation. J. Cell Sci. 108 (Pt 3), 1275–85. 7622610 10.1242/jcs.108.3.1275

[B64] Hwang J. H. Kim J. Y. Cha M. R. Ryoo I. J. Choo S. J. Cho S. M.. (2008). Etoposide-resistant HT-29 human colon carcinoma cells during glucose deprivation are sensitive to piericidin A, a GRP78 down-regulator. J. Cell. Physiol. 215, 243–250. 10.1002/jcp.21308 17941090

[B65] In J. Foulke-Abel J. Zachos N. C. Hansen A. M. Kaper J. B. Bernstein H. D.. (2016). Enterohemorrhagic *Escherichia coli* reduce mucus and intermicrovillar bridges in human stem cell-derived colonoids. Cell. Mol. Gastroenterol. Hepatol. 2, 48–62.e3. 10.1016/j.jcmgh.2015.10.001 26855967 PMC4740923

[B66] Isenmann R. Schwarz M. Rozdzinski E. Marre R. Beger H. G. (2000). Aggregation substance promotes colonic mucosal invasion of *Enterococcus faecalis* in an *ex vivo* model. J. Surg. Res. 89, 132–138. 10.1006/jsre.1999.5813 10729241

[B67] Jafari N. V. Kuehne S. A. Minton N. P. Allan E. Bajaj-Elliott M. (2016). Clostridium difficile-mediated effects on human intestinal epithelia: modelling host-pathogen interactions in a vertical diffusion chamber. Anaerobe 37, 96–102. 10.1016/j.anaerobe.2015.12.007 26708704

[B68] Jennis M. Cavanaugh C. R. Leo G. C. Mabus J. R. Lenhard J. Hornby P. J. (2018). Microbiota-derived tryptophan indoles increase after gastric bypass surgery and reduce intestinal permeability *in vitro* and *in vivo*. Neurogastroenterol. Motil. 30:e13178. 10.1111/nmo.13178 28782205

[B69] Jin Y. Blikslager A. T. (2016). Myosin light chain kinase mediates intestinal barrier dysfunction via occludin endocytosis during anoxia/reoxygenation injury. Am. J. Physiol. Cell Physiol. 311, C996–C1004. 10.1152/ajpcell.00113.2016 27760753

[B70] Johansson M. E. Phillipson M. Petersson J. Velcich A. Holm L. Hansson G. C. (2008). The inner of the two Muc2 mucin-dependent mucus layers in colon is devoid of bacteria. Proc. Natl. Acad. Sci. U.S.A. 105, 15064–15069. 10.1073/pnas.0803124105 18806221 PMC2567493

[B71] Kanehisa M. Goto S. Sato Y. Kawashima M. Furumichi M. Tanabe M. (2014). Data, information, knowledge and principle: back to metabolism in KEGG. Nucleic Acids Res. 42, D199–D205. 10.1093/nar/gkt1076 24214961 PMC3965122

[B72] Karl J. P. Hatch A. M. Arcidiacono S. M. Pearce S. C. Pantoja-Feliciano I. G. Doherty L. A.. (2018). Effects of psychological, environmental and physical stressors on the gut microbiota. Front. Microbiol. 9:2013. 10.3389/fmicb.2018.02013 30258412 PMC6143810

[B73] Karl J. P. Margolis L. M. Madslien E. H. Murphy N. E. Castellani J. W. Gundersen Y.. (2017a). Changes in intestinal microbiota composition and metabolism coincide with increased intestinal permeability in young adults under prolonged physiological stress. Am. J. Physiol. Gastrointest. Liver Physiol. 312, G559–G571. 10.1152/ajpgi.00066.2017 28336545

[B74] Karl J. P. Margolis L. M. Murphy N. E. Carrigan C. T. Castellani J. W. Madslien E. H.. (2017b). Military training elicits marked increases in plasma metabolomic signatures of energy metabolism, lipolysis, fatty acid oxidation, and ketogenesis. Physiol Rep 5:e13407. 10.14814/phy2.13407 28899914 PMC5599865

[B75] Karve S. S. Pradhan S. Ward D. V. Weiss A. A. (2017). Intestinal organoids model human responses to infection by commensal and Shiga toxin producing *Escherichia coli*. PLoS ONE 12:e0178966. 10.1371/journal.pone.0178966 28614372 PMC5470682

[B76] Kasendra M. Tovaglieri A. Sontheimer-Phelps A. Jalili-Firoozinezhad S. Bein A. Chalkiadaki A.. (2018). Development of a primary human small intestine-on-a-chip using biopsy-derived organoids. Sci. Rep. 8:2871. 10.1038/s41598-018-21201-7 29440725 PMC5811607

[B77] Khailova L. Mount Patrick S. K. Arganbright K. M. Halpern M. D. Kinouchi T. Dvorak B. (2010). Bifidobacterium bifidum reduces apoptosis in the intestinal epithelium in necrotizing enterocolitis. Am. J. Physiol. Gastrointest. Liver Physiol. 299, G1118–G1127. 10.1152/ajpgi.00131.2010 20705904 PMC2993641

[B78] Khan Mirzaei M. Haileselassie Y. Navis M. Cooper C. Sverremark-Ekstrom E. Nilsson A. S. (2016). Morphologically distinct *Escherichia coli* bacteriophages differ in their efficacy and ability to stimulate cytokine release *in vitro*. Front. Microbiol. 7:437 10.3389/fmicb.2016.00437 27065990 PMC4814447

[B79] Khanna K. Mishra K. P. Ganju L. Kumar B. Singh S. B. (2017). High-altitude-induced alterations in gut-immune axis: a review. Int. Rev. Immunol. 37, 119–126. 10.1080/08830185.2017.1407763 29231767

[B80] Khodaii Z. Ghaderian S. M. H. Natanzi M. M. (2017). Probiotic bacteria and their supernatants protect enterocyte cell lines from Enteroinvasive *Escherichia coli* (EIEC) invasion. Int. J. Mol. Cell. Med. 6, 183–189. 10.22088/acadpub.BUMS.6.3.183 29682490 PMC5898642

[B81] Kim H. J. Lee J. Choi J. H. Bahinski A. Ingber D. E. (2016a). Co-culture of living microbiome with microengineered human intestinal villi in a gut-on-a-chip microfluidic device. J. Vis. Exp. 114:e54344 10.3791/54344 PMC5091968 27684630

[B82] Kim H. J. Li H. Collins J. J. Ingber D. E. (2016b). Contributions of microbiome and mechanical deformation to intestinal bacterial overgrowth and inflammation in a human gut-on-a-chip. Proc. Natl. Acad. Sci. U.S.A. 113, E7–E15. 10.1073/pnas.1522193112 26668389 PMC4711860

[B83] Kinross J. M. Darzi A. W. Nicholson J. K. (2011). Gut microbiome-host interactions in health and disease. Genome Med. 3, 14–14. 10.1186/gm228 21392406 PMC3092099

[B84] Kleessen B. Schroedl W. Stueck M. Richter A. Rieck O. Krueger M. (2005). Microbial and immunological responses relative to high-altitude exposure in mountaineers. Med. Sci. Sports Exerc. 37, 1313–1318. 10.1249/01.mss.0000174888.22930.e0 16118577

[B85] Knight R. Vrbanac A. Taylor B. C. Aksenov A. Callewaert C. Debelius J.. (2018). Best practices for analysing microbiomes. Nat. Rev. Microbiol. 16, 410–422. 10.1038/s41579-018-0029-9 29795328

[B86] Knights D. Costello E. K. Knight R. (2011). Supervised classification of human microbiota. FEMS Microbiol. Rev. 35, 343–359. 10.1111/j.1574-6976.2010.00251.x 21039646

[B87] Lafuse W. P. Gearinger R. Fisher S. Nealer C. Mackos A. R. Bailey M. T. (2017). Exposure to a social stressor induces translocation of commensal lactobacilli to the spleen and priming of the innate immune system. J. Immunol. 198, 2383–2393. 10.4049/jimmunol.1601269 28167628 PMC5340647

[B88] Lambert G. P. (2008). Intestinal barrier dysfunction, endotoxemia, and gastrointestinal symptoms: the ‘canary in the coal mine' during exercise-heat stress? Med. Sport Sci. 53, 61–73. 10.1159/000151550 19208999

[B89] Larsen N. Nissen P. Willats W. G. (2007). The effect of calcium ions on adhesion and competitive exclusion of *Lactobacillus* ssp. and *E. coli* O138. Int. J. Food Microbiol. 114, 113–119. 10.1016/j.ijfoodmicro.2006.10.033 17234293

[B90] Le Bacquer O. Laboisse C. Darmaun D. (2003). Glutamine preserves protein synthesis and paracellular permeability in Caco-2 cells submitted to “luminal fasting.” Am. J. Physiol. Gastrointest. Liver Physiol. 285, G128–G136. 10.1152/ajpgi.00459.2002 12799310

[B91] LeBlanc J. G. Milani C. de Giori G. S. Sesma F. van Sinderen D. Ventura M. (2013). Bacteria as vitamin suppliers to their host: a gut microbiota perspective. Curr. Opin. Biotechnol. 24, 160–168. 10.1016/j.copbio.2012.08.005 22940212

[B92] Lee S. Y. Madan A. Furuta G. T. Colgan S. P. Sibley E. (2002). Lactase gene transcription is activated in response to hypoxia in intestinal epithelial cells. Mol. Genet. Metab. 75, 65–69. 10.1006/mgme.2001.3263 11825065

[B93] Lei Q. Qiang F. Chao D. Di W. Guoqian Z. Bo Y.. (2014). Amelioration of hypoxia and LPS-induced intestinal epithelial barrier dysfunction by emodin through the suppression of the NF-kappaB and HIF-1alpha signaling pathways. Int. J. Mol. Med. 34, 1629–1639. 10.3892/ijmm.2014.1965 25318952

[B94] Leslie J. L. Huang S. Opp J. S. Nagy M. S. Kobayashi M. Young V. B.. (2015). Persistence and toxin production by clostridium difficile within human intestinal organoids result in disruption of epithelial paracellular barrier function. Infect. Immun. 83, 138–145. 10.1128/IAI.02561-14 25312952 PMC4288864

[B95] Li J. Ayene R. Ward K. M. Dayanandam E. Ayene I. S. (2009). Glucose deprivation increases nuclear DNA repair protein Ku and resistance to radiation induced oxidative stress in human cancer cells. Cell Biochem. Funct. 27, 93–101. 10.1002/cbf.1541 19205005 PMC3145324

[B96] Li X. Kan E. M. Lu J. Cao Y. Wong R. K. Keshavarzian A.. (2013). Combat-training increases intestinal permeability, immune activation and gastrointestinal symptoms in soldiers. Aliment. Pharmacol. Therap. 37, 799–809. 10.1111/apt.12269 23432460

[B97] Li X. Wilder-Smith C. H. Kan M. E. Lu J. Cao Y. Wong R. K. (2014). Combat-training stress in soldiers increases S100B, a marker of increased blood-brain-barrier permeability, and induces immune activation. Neuro Endocrinol. Lett. 35, 58–63. 24625912

[B98] Li X. L. Peng Y. Z. Yuan Z. Q. Huang Y. S. Yang Z. C. (2003). Protection on proliferation of intestinal epithelial cells against hypoxia-reoxygenation through recombinant heat shock protein 70 adenovirus transfection. Zhongguo Wei Zhong Bing Ji Jiu Yi Xue 15, 81–83. 12857464

[B99] Li Y. Y. Ishihara S. Aziz M. M. Oka A. Kusunoki R. Tada Y.. (2011). Autophagy is required for toll-like receptor-mediated interleukin-8 production in intestinal epithelial cells. Int. J. Mol. Med. 27, 337–344. 10.3892/ijmm.2011.596 21225224

[B100] Lievin-Le Moal V. Servin A. L. (2013). Pathogenesis of human enterovirulent bacteria: lessons from cultured, fully differentiated human colon cancer cell lines. Microbiol. Mol. Biol. Rev. 77, 380–439. 10.1128/MMBR.00064-12 24006470 PMC3811612

[B101] Liu Y. Fatheree N. Y. Mangalat N. Rhoads J. M. (2010). Human-derived probiotic Lactobacillus reuteri strains differentially reduce intestinal inflammation. Am. J. Physiol. Gastrointest. Liver Physiol. 299, G1087–G1096. 10.1152/ajpgi.00124.2010 20798357 PMC2993169

[B102] Lukovac S. Belzer C. Pellis L. Keijser B. J. de Vos W. M. Montijn R. C.. (2014). Differential modulation by *Akkermansia muciniphila* and *Faecalibacterium prausnitzii* of host peripheral lipid metabolism and histone acetylation in mouse gut organoids. mBio 5:e01438-14. 10.1128/mBio.01438-14 25118238 PMC4145684

[B103] Maccaferri S. Klinder A. Cacciatore S. Chitarrari R. Honda H. Luchinat C.. (2012). *In vitro* fermentation of potential prebiotic flours from natural sources: impact on the human colonic microbiota and metabolome. Mol. Nutr. Food Res. 56, 1342–1352. 10.1002/mnfr.201200046 22753180

[B104] Macfarlane S. Macfarlane G. T. (2004). Bacterial diversity in the human gut. Adv. Appl. Microbiol. 54, 261–289. 10.1016/S0065-2164(04)54010-8 15251284

[B105] Mackos A. R. Maltz R. Bailey M. T. (2017). The role of the commensal microbiota in adaptive and maladaptive stressor-induced immunomodulation. Horm. Behav. 88, 70–78. 10.1016/j.yhbeh.2016.10.006 27760302 PMC5303636

[B106] Madara J. L. Dharmsathaphorn K. (1985). Occluding junction structure-function relationships in a cultured epithelial monolayer. J. Cell Biol. 101, 2124–2133. 10.1083/jcb.101.6.2124 3934178 PMC2114013

[B107] Manna C. Galletti P. Cucciolla V. Moltedo O. Leone A. Zappia V. (1997). The protective effect of the olive oil polyphenol (3,4-dihydroxyphenyl)-ethanol counteracts reactive oxygen metabolite-induced cytotoxicity in Caco-2 cells. J. Nutr. 127, 286–292. 10.1093/jn/127.2.286 9039829

[B108] Margolis L. M. Murphy N. E. Martini S. Gundersen Y. Castellani J. W. Karl J. P.. (2016). Effects of supplemental energy on protein balance during 4-d arctic military training. Med. Sci. Sports Exerc. 48, 1604–1612. 10.1249/MSS.0000000000000944 27054679

[B109] Margolis L. M. Murphy N. E. Martini S. Spitz M. G. Thrane I. McGraw S. M.. (2014). Effects of winter military training on energy balance, whole-body protein balance, muscle damage, soreness, and physical performance. Appl. Physiol. Nutr. Metab. 39, 1395–1401. 10.1139/apnm-2014-0212 25386980

[B110] Mariani V. Palermo S. Fiorentini S. Lanubile A. Giuffra E. (2009). Gene expression study of two widely used pig intestinal epithelial cell lines: IPEC-J2 and IPI-2I. Vet. Immunol. Immunopathol. 131, 278–284. 10.1016/j.vetimm.2009.04.006 19446887

[B111] Mayer E. A. Tillisch K. Gupta A. (2015). Gut/brain axis and the microbiota. J. Clin. Invest. 125, 926–938. 10.1172/JCI76304 25689247 PMC4362231

[B112] McCabe R. E. Yu G. S. Conteas C. Morrill P. R. McMorrow B. (1991). *In vitro* model of attachment of Giardia intestinalis trophozoites to IEC-6 cells, an intestinal cell line. Antimicrob. Agents Chemother. 35, 29–35. 10.1128/AAC.35.1.29 1901700 PMC244937

[B113] McOrist S. Jasni S. Mackie R. A. Berschneider H. M. Rowland A. C. Lawson G. H. (1995). Entry of the bacterium ileal symbiont intracellularis into cultured enterocytes and its subsequent release. Res. Vet. Sci. 59, 255–260. 10.1016/0034-5288(95)90013-6 8588102

[B114] Miki K. Unno N. Nagata T. Uchijima M. Konno H. Koide Y.. (2004). Butyrate suppresses hypoxia-inducible factor-1 activity in intestinal epithelial cells under hypoxic conditions. Shock 22, 446–452. 10.1097/01.shk.0000140664.80530.bd 15489637

[B115] Montain S. J. Young A. J. (2003). Diet and physical performance. Appetite 40, 255–267. 10.1016/S0195-6663(03)00011-4 12798783

[B116] Mowat A. M. (2003). Anatomical basis of tolerance and immunity to intestinal antigens. Nat. Rev. Immunol. 3, 331–341. 10.1038/nri1057 12669023

[B117] Mukherjee P. Mani S. (2013). Methodologies to decipher the cell secretome. Biochim. Biophys. Acta 1834, 2226–2232. 10.1016/j.bbapap.2013.01.022 23376189 PMC3652893

[B118] Naito T. Mulet C. De Castro C. Molinaro A. Saffarian A. Nigro G.. (2017). Lipopolysaccharide from crypt-specific core microbiota modulates the colonic epithelial proliferation-to-differentiation balance. mBio 8:e01680-17. 10.1128/mBio.01680-17 29042502 PMC5646255

[B119] Nepelska M. Cultrone A. Beguet-Crespel F. Le Roux K. Dore J. Arulampalam V.. (2012). Butyrate produced by commensal bacteria potentiates phorbol esters induced AP-1 response in human intestinal epithelial cells. PLoS ONE 7:e52869. 10.1371/journal.pone.0052869 23300800 PMC3531367

[B120] Nguyen T. L. Vieira-Silva S. Liston A. Raes J. (2015). How informative is the mouse for human gut microbiota research? Dis. Model Mech. 8, 1–16. 10.1242/dmm.017400 25561744 PMC4283646

[B121] Nigro G. Rossi R. Commere P. H. Jay P. Sansonetti P. J. (2014). The cytosolic bacterial peptidoglycan sensor Nod2 affords stem cell protection and links microbes to gut epithelial regeneration. Cell Host Microbe 15, 792–798. 10.1016/j.chom.2014.05.003 24882705

[B122] Nindl B. C. Castellani J. W. Warr B. J. Sharp M. A. Henning P. C. Spiering B. A.. (2013). Physiological employment standards III: physiological challenges and consequences encountered during international military deployments. Eur. J. Appl. Physiol. 113, 2655–2672. 10.1007/s00421-013-2591-1 23430237

[B123] Noel G. Baetz N. W. Staab J. F. Donowitz M. Kovbasnjuk O. Pasetti M. F. (2017). A primary human macrophage-enteroid co-culture model to investigate mucosal gut physiology and host-pathogen interactions. Sci. Rep. 7:45270 10.1038/srep45270 28345602 PMC5366908

[B124] Osborne D. L. Seidel E. R. (1989). Microflora-derived polyamines modulate obstruction-induced colonic mucosal hypertrophy. Am. J. Physiol. 256, G1049–G1057. 10.1152/ajpgi.1989.256.6.G1049 2544100

[B125] Pan F. Han L. Zhang Y. Yu Y. Liu J. (2015). Optimization of Caco-2 and HT29 co-culture *in vitro* cell models for permeability studies. Int. J. Food Sci. Nutr. 66, 680–685. 10.3109/09637486.2015.1077792 26299896

[B126] Park J. H. Kotani T. Konno T. Setiawan J. Kitamura Y. Imada S.. (2016). Promotion of intestinal epithelial cell turnover by commensal bacteria: role of short-chain fatty acids. PLoS ONE 11:e0156334. 10.1371/journal.pone.0156334 27232601 PMC4883796

[B127] Pearce S. C. Al-Jawadi A. Kishida K. Yu S. Hu M. Fritzky L. F. (2018). Marked differences in tight junction composition and macromolecular permeability among different intestinal cell types. BMC Biol. 16:19 10.1186/s12915-018-0481-z 29391007 PMC5793346

[B128] Pearce S. C. Mani V. Weber T. E. Rhoads R. P. Patience J. F. Baumgard L. H.. (2013). Heat stress and reduced plane of nutrition decreases intestinal integrity and function in pigs. J. Anim. Sci. 91, 5183–5193. 10.2527/jas.2013-6759 23989867

[B129] Pearce S. C. Sanz-Fernandez M. V. Hollis J. H. Baumgard L. H. Gabler N. K. (2014). Short-term exposure to heat stress attenuates appetite and intestinal integrity in growing pigs. J. Anim. Sci. 92, 5444–5454. 10.2527/jas.2014-8407 25367514

[B130] Phua L. C. Wilder-Smith C. H. Tan Y. M. Gopalakrishnan T. Wong R. K. Li X.. (2015). Gastrointestinal symptoms and altered intestinal permeability induced by combat training are associated with distinct metabotypic changes. J. Proteome Res. 14, 4734–4742. 10.1021/acs.jproteome.5b00603 26506213

[B131] Pierzchalska M. Panek M. Czyrnek M. Gielicz A. Mickowska B. Grabacka M. (2017). Probiotic *Lactobacillus acidophilus* bacteria or synthetic TLR2 agonist boost the growth of chicken embryo intestinal organoids in cultures comprising epithelial cells and myofibroblasts. Comp. Immunol. Microbiol. Infect. Dis. 53, 7–18. 10.1016/j.cimid.2017.06.002 28750869

[B132] Pompaiah M. Bartfeld S. (2017). Gastric organoids: an emerging model system to study *Helicobacter pylori* pathogenesis. Curr. Top. Microbiol. Immunol. 400, 149–168. 10.1007/978-3-319-50520-6_7 28124153

[B133] Porter C. K. Olson S. Hall A. Riddle M. S. (2017). Travelers' diarrhea: an update on the incidence, etiology, and risk in military deployments and similar travel populations. Mil. Med. 182, 4–10. 10.7205/MILMED-D-17-00064 28885918

[B134] Raja S. B. Murali M. R. Devaraj H. Devaraj S. N. (2012). Differential expression of gastric MUC5AC in colonic epithelial cells: TFF3-wired IL1 beta/Akt crosstalk-induced mucosal immune response against *Shigella dysenteriae* infection. J. Cell Sci. 125, 703–713. 10.1242/jcs.092148 22389405

[B135] Rajan A. Vela L. Zeng X. L. Yu X. Shroyer N. Blutt S. E.. (2018). Novel segment- and host-specific patterns of enteroaggregative *Escherichia coli* adherence to human intestinal enteroids. mBio 9:e02419-17. 10.1128/mBio.02419-17 29463660 PMC5821088

[B136] Resta-Lenert S. Barrett K. E. (2003). Live probiotics protect intestinal epithelial cells from the effects of infection with enteroinvasive *Escherichia coli* (EIEC). Gut 52, 988–997. 10.1136/gut.52.7.988 12801956 PMC1773702

[B137] Rhoads J. M. Chen W. Chu P. Berschneider H. M. Argenzio R. A. Paradiso A. M. (1994). L-glutamine and L-asparagine stimulate Na^+^-H^+^ exchange in porcine jejunal enterocytes. Am. J. Physiol. 266, G828–G838. 10.1152/ajpgi.1994.266.5.G828 8203529

[B138] Riddle M. S. Martin G. J. Murray C. K. Burgess T. H. Connor P. Mancuso J. D.. (2017). Management of acute diarrheal illness during deployment: a deployment health guideline and expert panel report. Mil. Med. 182, 34–52. 10.7205/MILMED-D-17-00077 28885922 PMC5657341

[B139] Roeselers G. Ponomarenko M. Lukovac S. Wortelboer H. M. (2013). *Ex vivo* systems to study host–microbiota interactions in the gastrointestinal tract. Best Pract. Res. Clin. Gastroenterol. 27, 101–113. 10.1016/j.bpg.2013.03.018 23768556

[B140] Rothschild D. E. Srinivasan T. Aponte-Santiago L. A. Shen X. Allen I. C. (2016). The *ex vivo* culture and pattern recognition receptor stimulation of mouse intestinal organoids. J. Vis. Exp. 111:e54033 10.3791/54033 PMC4927701 27285214

[B141] Round J. L. O'Connell R. M. Mazmanian S. K. (2010). Coordination of tolerogenic immune responses by the commensal microbiota. J. Autoimmun. 34, J220–J225. 10.1016/j.jaut.2009.11.007 19963349 PMC3155383

[B142] Roussel G. Stevens V. Cottin S. McArdle H. J. (2017). The effect of amino acid deprivation on the transfer of iron through Caco-2 cell monolayers. J. Trace Elem. Med. Biol. 40, 82–90. 10.1016/j.jtemb.2016.12.016 28159226

[B143] Rousset M. (1986). The human colon carcinoma cell lines HT-29 and Caco-2: two *in vitro* models for the study of intestinal differentiation. Biochimie 68, 1035–1040. 10.1016/S0300-9084(86)80177-8 3096381

[B144] Russell W. R. Hoyles L. Flint H. J. Dumas M. E. (2013). Colonic bacterial metabolites and human health. Curr. Opin. Microbiol. 16, 246–254. 10.1016/j.mib.2013.07.002 23880135

[B145] Saito Y. Iwatsuki K. Hanyu H. Maruyama N. Aihara E. Tadaishi M.. (2017). Effect of essential amino acids on enteroids: methionine deprivation suppresses proliferation and affects differentiation in enteroid stem cells. Biochem. Biophys. Res. Commun. 488, 171–176. 10.1016/j.bbrc.2017.05.029 28483523

[B146] Saravi F. D. Chirino D. R. Saldena T. A. Cincunegui L. M. Carra G. E. Ituarte L. M. (2002). Chronic hypobaric hypoxia effects on rat colon in vitro sensitivity to acute hypoxia and amiloride. Digest. Dis. Sci. 47, 1086–1090. 10.1023/A:1015094225062 12018904

[B147] Sato T. Vries R. G. Snippert H. J. van de Wetering M. Barker N. Stange D. E.. (2009). Single Lgr5 stem cells build crypt-villus structures *in vitro* without a mesenchymal niche. Nature 459, 262–265. 10.1038/nature07935 19329995

[B148] Saunders P. R. Kosecka U. McKay D. M. Perdue M. H. (1994). Acute stressors stimulate ion secretion and increase epithelial permeability in rat intestine. Am. J. Physiol. 267, G794–G799. 10.1152/ajpgi.1994.267.5.G794 7977741

[B149] Schierack P. Nordhoff M. Pollmann M. Weyrauch K. D. Amasheh S. Lodemann U.. (2006). Characterization of a porcine intestinal epithelial cell line for *in vitro* studies of microbial pathogenesis in swine. Histochem. Cell Biol. 125, 293–305. 10.1007/s00418-005-0067-z 16215741

[B150] Schilderink R. Verseijden C. Seppen J. Muncan V. van den Brink G. R. Lambers T. T.. (2016). The SCFA butyrate stimulates the epithelial production of retinoic acid via inhibition of epithelial HDAC. Am. J. Physiol. Gastrointest. Liver Physiol. 310, G1138–46. 10.1152/ajpgi.00411.2015 27151945

[B151] Schmid S. M. Hallschmid M. Schultes B. (2015). The metabolic burden of sleep loss. Lancet Diabetes Endocrinol. 3, 52–62. 10.1016/S2213-8587(14)70012-9 24731536

[B152] Segata N. Boernigen D. Tickle T. L. Morgan X. C. Garrett W. S. Huttenhower C. (2013). Computational meta'omics for microbial community studies. Mol. Syst. Biol. 9:666. 10.1038/msb.2013.22 23670539 PMC4039370

[B153] Shah P. Fritz J. V. Glaab E. Desai M. S. Greenhalgh K. Frachet A.. (2016). A microfluidics-based *in vitro* model of the gastrointestinal human-microbe interface. Nat. Commun. 7:11535. 10.1038/ncomms11535 27168102 PMC4865890

[B154] Shi C. Z. Chen H. Q. Liang Y. Xia Y. Yang Y. Z. Yang J.. (2014). Combined probiotic bacteria promotes intestinal epithelial barrier function in interleukin-10-gene-deficient mice. World J. Gastroenterol. 20, 4636–4647. 10.3748/wjg.v20.i16.4636 24782616 PMC4000500

[B155] Shi Y. H. Xiao J. J. Feng R. P. Liu Y. Y. Liao M. Wu X. W.. (2017). Factors affecting the bioaccessibility and intestinal transport of difenoconazole, hexaconazole, and spirodiclofen in human Caco-2 cells following *in vitro* digestion. J. Agric. Food Chem. 65, 9139–9146. 10.1021/acs.jafc.7b02781 28915046

[B156] Skjolaas K. A. Burkey T. E. Dritz S. S. Minton J. E. (2007). Effects of *Salmonella enterica* serovar *Typhimurium*, or serovar *Choleraesuis, Lactobacillus reuteri* and *Bacillus licheniformis* on chemokine and cytokine expression in the swine jejunal epithelial cell line, IPEC-J2. Vet. Immunol. Immunopathol. 115, 299–308. 10.1016/j.vetimm.2006.10.012 17157391

[B157] Smirnova M. G. Guo L. Birchall J. P. Pearson J. P. (2003). LPS up-regulates mucin and cytokine mRNA expression and stimulates mucin and cytokine secretion in goblet cells. Cell Immunol. 221, 42–49. 10.1016/S0008-8749(03)00059-5 12742381

[B158] Sommer F. Anderson J. M. Bharti R. Raes J. Rosenstiel P. (2017). The resilience of the intestinal microbiota influences health and disease. Nat. Rev. Microbiol. 15, 630–638. 10.1038/nrmicro.2017.58 28626231

[B159] Swank G. M. Lu Q. Xu D. Z. Michalsky M. Deitch E. A. (1998). Effect of acute-phase and heat-shock stress on apoptosis in intestinal epithelial cells (Caco-2). Crit. Care Med. 26, 1213–1217. 10.1097/00003246-199807000-00023 9671371

[B160] Thaiss C. A. Levy M. Korem T. Dohnalova L. Shapiro H. Jaitin D. A.. (2016). Microbiota diurnal rhythmicity programs host transcriptome oscillations. Cell 167, 1495–1510.e12. 10.1016/j.cell.2016.11.003 27912059

[B161] Tharion W. J. Lieberman H. R. Montain S. J. Young A. J. Baker-Fulco C. J. Delany J. P.. (2005). Energy requirements of military personnel. Appetite 44, 47–65. 10.1016/j.appet.2003.11.010 15604033

[B162] Toki S. Kagaya S. Shinohara M. Wakiguchi H. Matsumoto T. Takahata Y.. (2009). *Lactobacillus rhamnosus* GG and *Lactobacillus casei* suppress *Escherichia coli*-induced chemokine expression in intestinal epithelial cells. Int. Arch. Allergy Immunol. 148, 45–58. 10.1159/000151505 18716403

[B163] Townsend J. H. Davis S. R. Mackey A. D. Gregory J. F. I. I. I. (2004). Folate deprivation reduces homocysteine remethylation in a human intestinal epithelial cell culture model: role of serine in one-carbon donation. Am. J. Physiol. Gastrointest. Liver Physiol. 286, G588–G595. 10.1152/ajpgi.00454.2003 14615285

[B164] Trapecar M. Goropevsek A. Gorenjak M. Gradisnik L. Slak Rupnik M. (2014). A co-culture model of the developing small intestine offers new insight in the early immunomodulation of enterocytes and macrophages by *Lactobacillus* spp. through STAT1 and NF-kB p65 translocation. PLoS ONE 9:e86297. 10.1371/journal.pone.0086297 24454965 PMC3894201

[B165] Unno N. Menconi M. J. Salzman A. L. Smith M. Hagen S. Ge Y.. (1996). Hyperpermeability and ATP depletion induced by chronic hypoxia or glycolytic inhibition in Caco-2BBe monolayers. Am. J. Physiol. 270, G1010–G1021. 10.1152/ajpgi.1996.270.6.G1010 8764209

[B166] Van den Abbeele P. Belzer C. Goossens M. Kleerebezem M. De Vos W. M. Thas O.. (2013). Butyrate-producing clostridium cluster XIVa species specifically colonize mucins in an *in vitro* gut model. ISME J. 7, 949–961. 10.1038/ismej.2012.158 23235287 PMC3635240

[B167] van Kooyk Y. (2008). C-type lectins on dendritic cells: key modulators for the induction of immune responses. Biochem. Soc. Trans. 36, 1478–1481. 10.1042/BST0361478 19021579

[B168] Voigt R. M. Forsyth C. B. Green S. J. Engen P. A. Keshavarzian A. (2016). Circadian rhythm and the gut microbiome. Int. Rev. Neurobiol. 131, 193–205. 10.1016/bs.irn.2016.07.002 27793218

[B169] Wang H. Bastian S. E. Cheah K. Y. Lawrence A. Howarth G. S. (2014). *Escherichia coli* nissle 1917-derived factors reduce cell death and late apoptosis and increase transepithelial electrical resistance in a model of 5-fluorouracil-induced intestinal epithelial cell damage. Cancer Biol. Ther. 15, 560–569. 10.4161/cbt.28159 24556751 PMC4026078

[B170] Weeks S. R. McAuliffe C. L. Durussel D. Pasquina P. F. (2010). Physiological and psychological fatigue in extreme conditions: the military example. PM R. 2, 438–441. 10.1016/j.pmrj.2010.03.023 20656625

[B171] Winter S. E. Baumler A. J. (2014). Why related bacterial species bloom simultaneously in the gut: principles underlying the ‘Like will to like' concept. Cell Microbiol. 16, 179–184. 10.1111/cmi.12245 24286560 PMC4013256

[B172] Wlodarska M. Luo C. Kolde R. d'Hennezel E. Annand J. W. Heim C. E.. (2017). Indoleacrylic acid produced by commensal peptostreptococcus species suppresses inflammation. Cell Host Microbe 22, 25–37.e6. 10.1016/j.chom.2017.06.007 28704649 PMC5672633

[B173] Workman M. J. Gleeson J. P. Troisi E. J. Estrada H. Q. Kerns S. J. Hinojosa C. D.. (2018). Enhanced utilization of induced pluripotent stem cell-derived human intestinal organoids using microengineered chips. Cell. Mol. Gastroenterol. Hepatol. 5, 669–677.e2. 10.1016/j.jcmgh.2017.12.008 29930984 PMC6009013

[B174] Xiao G. Tang L. Yuan F. Zhu W. Zhang S. Liu Z.. (2013). Eicosapentaenoic acid enhances heat stress-impaired intestinal epithelial barrier function in Caco-2 cells. PLoS ONE 8:e73571. 10.1371/journal.pone.0073571 24066055 PMC3774713

[B175] Xu D. Z. Lu Q. Kubicka R. Deitch E. A. (1999). The effect of hypoxia/reoxygenation on the cellular function of intestinal epithelial cells. J. Trauma 46, 280–285. 10.1097/00005373-199902000-00014 10029034

[B176] Xu D. Z. Lu Q. Swank G. M. Deitch E. A. (1996). Effect of heat shock and endotoxin stress on enterocyte viability apoptosis and function varies based on whether the cells are exposed to heat shock or endotoxin first. Arch. Surg. 131, 1222–1228. 10.1001/archsurg.1996.01430230104018 8911264

[B177] Yang X. Gao X. C. Liu J. Ren H. Y. (2017). Effect of EPEC endotoxin and bifidobacteria on intestinal barrier function through modulation of toll-like receptor 2 and toll-like receptor 4 expression in intestinal epithelial cell-18. World J. Gastroenterol. 23, 4744–4751. 10.3748/wjg.v23.i26.4744 28765695 PMC5514639

[B178] Yazdani B. C. T. M. Debelius J. W. Li W. Knight R. Smarr L. (2016). Using machine learning to identify major shifts in human gut microbiome protein family abundance in disease, in 2016 IEEE International Conference on Big Data (Big Data) (San Diego, CA), 1272–1280.

[B179] Yin X. Farin H. F. van Es J. H. Clevers H. Langer R. Karp J. M. (2014). Niche-independent high-purity cultures of Lgr5^+^ intestinal stem cells and their progeny. Nat. Methods 11, 106–112. 10.1038/nmeth.2737 24292484 PMC3951815

[B180] Yoo B. B. Mazmanian S. K. (2017). The enteric network: interactions between the immune and nervous systems of the gut. Immunity 46, 910–926. 10.1016/j.immuni.2017.05.011 28636959 PMC5551410

[B181] Yu J. Liu F. Yin P. Zhao H. Luan W. Hou X.. (2013). Involvement of oxidative stress and mitogen-activated protein kinase signaling pathways in heat stress-induced injury in the rat small intestine. Stress 16, 99–113. 10.3109/10253890.2012.680526 22452662

[B182] Zeitouni N. E. Dersch P. Naim H. Y. von Kockritz-Blickwede M. (2016). Hypoxia decreases invasin-mediated yersinia enterocolitica internalization into Caco-2 cells. PLoS ONE 11:e0146103. 10.1371/journal.pone.0146103 26731748 PMC4701670

[B183] Zenhom M. Hyder A. de Vrese M. Heller K. J. Roeder T. Schrezenmeir J. (2011). Prebiotic oligosaccharides reduce proinflammatory cytokines in intestinal Caco-2 cells via activation of PPARgamma and peptidoglycan recognition protein 3. J. Nutr. 141, 971–977. 10.3945/jn.110.136176 21451128

[B184] Zhang Y. G. Wu S. Xia Y. Sun J. (2014). Salmonella-infected crypt-derived intestinal organoid culture system for host-bacterial interactions. Physiol. Rep. 2:e12147. 10.14814/phy2.12147 25214524 PMC4270227

[B185] Zhou Q. Q. Yang D. Z. Luo Y. J. Li S. Z. Liu F. Y. Wang G. S. (2011). Over-starvation aggravates intestinal injury and promotes bacterial and endotoxin translocation under high-altitude hypoxic environment. World J. Gastroenterol. 17, 1584–1593. 10.3748/wjg.v17.i12.1584 21472125 PMC3070130

[B186] Zhu A. Sunagawa S. Mende D. R. Bork P. (2015). Inter-individual differences in the gene content of human gut bacterial species. Genome Biol. 16:82. 10.1186/s13059-015-0646-9 25896518 PMC4428241

[B187] Zhu X. Han Y. Du J. Liu R. Jin K. Yi W. (2017). Microbiota-gut-brain axis and the central nervous system. Oncotarget 8, 53829–53838. 10.18632/oncotarget.17754 28881854 PMC5581153

[B188] Zietak M. Kovatcheva-Datchary P. Markiewicz L. H. Stahlman M. Kozak L. P. Backhed F. (2016). Altered microbiota contributes to reduced diet-induced obesity upon cold exposure. Cell Metab. 23, 1216–1223. 10.1016/j.cmet.2016.05.001 27304513 PMC4911343

[B189] Zomer-van Ommen D. D. Pukin A. V. Fu O. Quarles van Ufford L. H. Janssens H. M. Beekman J. M.. (2016). Functional characterization of cholera toxin inhibitors using human intestinal organoids. J. Med. Chem. 59, 6968–6972. 10.1021/acs.jmedchem.6b00770 27347611

[B190] Zuhl M. Schneider S. Lanphere K. Conn C. Dokladny K. Moseley P. (2014). Exercise regulation of intestinal tight junction proteins. Br. J. Sports Med. 48, 980–986. 10.1136/bjsports-2012-091585 23134759

